# Clearance of therapeutic antibody glycoforms after subcutaneous and intravenous injection in a porcine model

**DOI:** 10.1080/19420862.2022.2145929

**Published:** 2022-11-16

**Authors:** David Falck, Martin Lechmann, Ana Momčilović, Marco Thomann, Carolien A. M. Koeleman, Cordula Jany, Sebastian Malik, Manfred Wuhrer, Dietmar Reusch

**Affiliations:** a Center for Proteomics and Metabolomics, Leiden University Medical Center, Leiden, The Netherlands; b Roche Pharma Research and Early Development, Pharmaceutical Sciences, Roche Innovation Center Munich, Penzberg, Germany; c Pharma Technical Development Europe, Roche Diagnostics GmbH, Penzberg, Germany

**Keywords:** Pharmacokinetics, N-glycosylation, monoclonal antibodies, liquid chromatography – mass spectrometry, glycoengineering, minipig, subcutaneous injection

## Abstract

A relatively low clearance is one of the prominent favorable features of immunoglobulin G1-based therapeutic monoclonal antibodies (mAbs). Various studies have observed differential clearance of mAb glycoforms, including oligomannose glycoforms, which are considered a critical quality attribute because they show higher clearance than complex type glycoforms. Glycoform clearance, however, has not previously been studied after subcutaneous injection or in a porcine model system. Here, we performed glycoform-resolved pharmacokinetic (PK) analysis of two mAbs in Göttingen minipigs. We found glycoform effects on clearance to be largely the same for subcutaneous and intravenous injection and in line with observations in other species. Oligomannose glycoforms were cleared up to 25% faster and monoantennary glycoforms up to 8% faster than agalactosylated complex glycoforms. Sialylated glycoforms were cleared at approximately the same rate as fully galactosylated glycoforms. Importantly, we report here an impact of galactosylation on the PK of a mAb for the first time. Whether increased galactosylation led to slower or faster clearance seemed to depend on the overall glycosylation profile. When clearance of galactosylated glycoforms was slower, the mAb showed higher galactosylation in serum at maximum concentration after subcutaneous injection compared to both intravenous injection and the injected material. Whether this higher galactosylation after subcutaneous injection has consequences for therapeutic efficacy remains to be investigated. In conclusion, preferential clearance of antibody glycoforms can be simulated in the minipig model with intravenous as well as subcutaneous injections. Furthermore, we observed a glycoform bias in the absorption from skin into circulation after subcutaneous injection based on galactosylation.

**Abbreviations**: AUC - area under the curve; CL/F - apparent clearance as a function of bioavailability following SC administration; C_max_ - maximum serum concentration; CQA critical quality attribute; FcγR - Fc gamma receptor; IgG - immunoglobulin G; IV - intravenous; LC-MS - liquid chromatography - mass spectrometry; mAb - therapeutic monoclonal antibody; PK - pharmacokinetics; SC - subcutaneous; TMDD - target-mediated drug disposition

## Introduction

Pharmacokinetics (PK) of biopharmaceuticals strongly influence their efficacy. Therapeutic monoclonal antibodies (mAbs) are widely used and highly efficacious drugs, but they are also costly and usually rely on injectable formulations.^1–3^ Thus, every change in dose and dosing frequency can have effects on healthcare system sustainability and patient comfort.^4^ MAb glycosylation is a critical quality attribute (CQA) which is thoroughly controlled. Glycosylation is regularly designed to control effector functions, such as antibody-dependent cell-mediated cytotoxicity (ADCC).^5^ However, the impact of mAb glycosylation on PK behavior remains understudied, and therefore prone to misrepresentation in CQA assessments.^6^

Oligomannose and hybrid-type glycans have been shown to reduce the half-life and increase the clearance of immunoglobulin G (IgG)-based mAbs.^7^ Oligomannose and hybrid-type glycans share outer arm mannoses as a structural motif, suggesting that these outer arm mannoses are mediators of faster clearance. Monoantennary glycans, which feature a terminal mannose but lack outer arm mannoses, also promote faster clearance, but are three to 10 times less impactful in this respect.^8^ Glycans in the Fab domain of mAbs or the receptor domain of fusion proteins may have a larger and more diverse impact on PK than Fc-glycans of mAbs.^9^ A detailed overview of the existing knowledge can be found elsewhere.^6,8^ The PK impact of mAb glycosylation has been studied in rodents, rabbits and monkeys, as well as in human Phase 1 clinical studies. However, no studies in minipigs have been reported, although these animals represent an important alternative to monkey models. Because of increased ethical concerns regarding the use of primates in non-clinical testing, attention has focused on the potential use of minipigs as non-rodent alternatives for pharmaceutical testing.^10,11^ There is increasing evidence demonstrating similarities between pig/minipig and human skin and lymph architecture, which are main contributors to subcutaneous (SC) absorption and bioavailability of macromolecules.^12,13^ In addition, the thickness of the epidermis and the stratum corneum, as well as the lipid composition of the stratum corneum, show many similarities between human and pigs. Therefore, the minipig model is frequently used for dermal safety testing.^14^ All of this makes minipigs preferred models for exploring SC administration routes, with advantages over non-human primates.^15^

SC dosing of biologics is desirable,^16^ as it offers several advantages over IV administration, such as fixed dosing, lower hospital and clinical costs, and increased patient throughput.^3,5^ In addition, SC is typically more convenient for patients compared with IV delivery because it reduces administration time and allows for self-administered or caregiver-supported dosing at home, thereby reducing treatment burden and improving quality of life.^3,5,6^ The delayed release of therapeutic antibodies into circulation may lead to more uniform serum concentration over time and can be used to mitigate maximum serum concentration (*C_max_*)-driven site effects, such as cytokine release syndrome.^17^

Lysosomal degradation in endothelial and hematopoietic cells is the main clearance route for mAbs and largely fed by nonspecific pinocytosis.^4,18^ However, target-mediated drug disposition (TMDD) may also contribute to clearance.^19^ Since immune cells may contribute to TMDD, the potential influence of mAb Fc glycosylation on immune activation needs to be considered in interpreting mAb clearance.^19,20^

**Figure 1.**
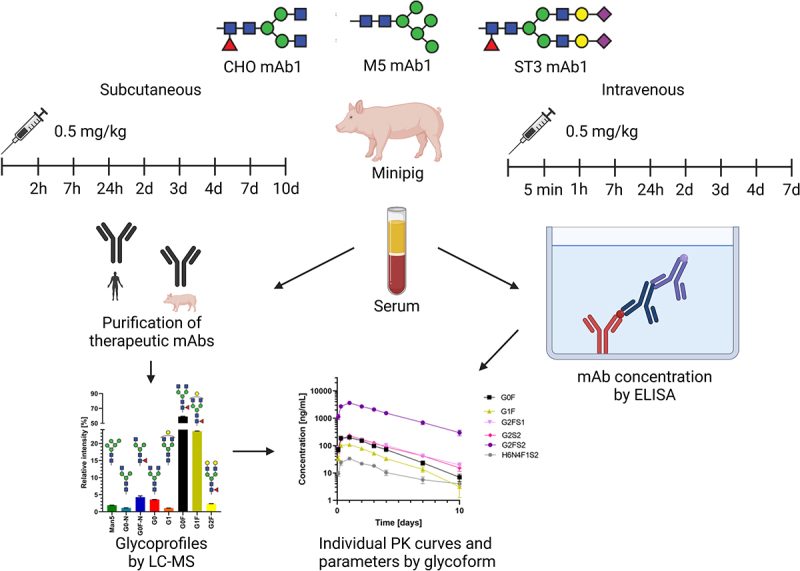
Overview of animal experiments and analytical workflow.

Herein, we report the glycoform-resolved PK analysis of two mAbs in Göttingen minipigs (Sus scrofa domesticus). The study focuses on mAb1, a therapeutic mAb used in oncology. In particular, we contrasted the effects of glycosylation on PK behavior between IV and SC delivery (Figure 1). MAb1 showed non-linear pharmacokinetics in minipigs, which is indicative of target being present. Therefore, we also investigated mAb2, which has neither a target in pigs nor any Fc gamma receptor affinity. The comparison between mAb1 and mAb2 should highlight potential effects of TMDD. Glycoform-resolved PK analysis was achieved as previously reported, combining absolute mAb concentrations measured by enzyme-linked immunosorbent assay (ELISA) and time-dependent glycosylation profiles measured by liquid chromatography – mass spectrometry (LC-MS; Figure 1).^8^ To enhance the glycoform-resolved PK studies, we used glycoengineered versions of the mAbs. These mainly carried either a Man5 oligomannose glycan (M5) or a fully galactosylated and α2,3-sialylated diantennary complex glycan (ST3), in addition to the mAbs with typical Chinese hamster ovary (CHO) cell type glycosylation. Through choosing a glycopeptide-based method for the analysis of mAb glycosylation, information on glycan pairing is lost. This has to be considered when comparing PK parameters between experiments, as they will be dominated by the major glycan presenting the likely pairing partner for other glycans. While this glycoform pairing bias may lead to differences between antibody preparations, glycoforms within a single preparation may be assumed to have the same partner, thus canceling the pairing bias. Therefore, qualitative and quantitative comparisons of glycoform PK parameters within an experiment are valid.^6,8^

## Results

**Figure 2.**
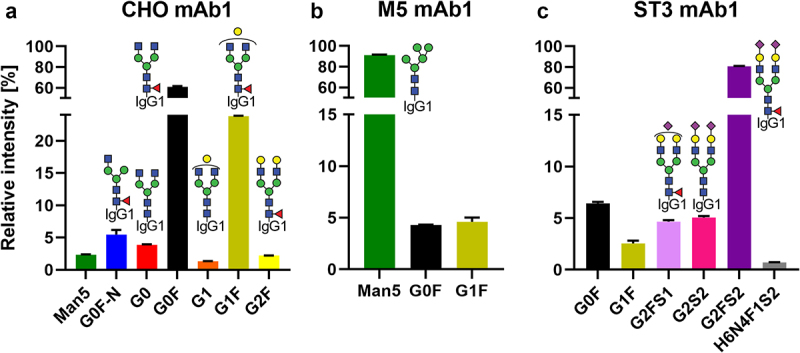
Relative abundance of quantified glycoforms in the mAb1 starting materials. For respective information on mAb2, see Figure S1. Further information on glycoform nomenclature can be found in Table S1.

The three versions of mAb1 showed the desired glycosylation profiles (see Table S1 for nomenclature). CHO mAb1 consisted mainly of fucosylated diantennary glycans of the complex type with varying degrees of terminal galactosylation (Figure 2a). Minor quantities of oligomannose, fucosylated monoantennary or afucosylated dianntenary glycans were observed as well. The M5 mAb1 was dominated by the oligomannose glycoform Man5, showing only minor amounts of fucosylated diantennary glycans with none or one galactose (Figure 2b). The ST3 mAb1 was dominated by a fully elaborated diantennary glycan (G2FS2 ca. 80%), with minor contributions of mono- and asialylated, mono- and agalactosylated as well as afucosylated forms (Figure 2c). For mAb2, a CHO and an ST3 version were used. CHO mAb2 had the same glycoforms as CHO mAb1, albeit with a significantly more elaborated galactosylation (Figure1a). ST3 mAb2 was also similar to ST3 mAb1, with higher galactosylation, but less sialylation. However, ST3 mAb2 differed from the ST3 mAb1 in that it contained discernable amounts of oligomannose and monoantennary glycans (Figure S1B).

**Figure 3.**
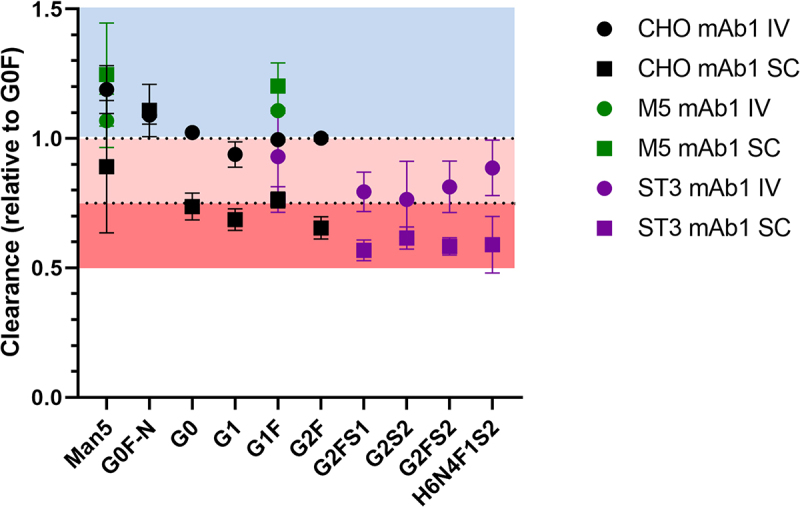
Clearance of the individual glycoforms of the three mAb1 glycovariants, normalized to the clearance rate of G0F in each animal. Mean and 95% confidence interval are depicted; n = 5. Standard deviation ranged from 0.9% to 23%.

Regarding glycoform-resolved PK, the combination of the glycosylation profiles (Table S2) and the absolute mAb concentrations (Table S3) at different time points resulted in individual glycoform concentrations (Table S3). From the individual concentrations, PK parameters were calculated separately, revealing differences between the individual glycoforms (Table 1, Figure 3 and Tables S4 to S11). Though later time points were available for the same animals, we limited our analysis of the mAb1 PK studies to those samples where glycosylation could reliably be determined. We also chose a maximum of 10 d to minimize interferences from anti-drug antibodies which started to appear a week after SC injection in the majority of animals (data not shown). Antibodies with the oligomannose glycan Man5 were cleared faster than the agalactosylated and fucosylated complex glycoform (G0F), which served as a reference. This effect was also seen in mAb2 (Tables S10 and S11). Monoantennary glycoforms were also cleared faster, as seen for the G0F-N glycan in CHO mAb1. The G1S-N and G1FS-N glycans in ST3 mAb2 also showed faster clearance as compared to G2F, which we chose as reference in ST3 mAb2 due to the absence of G0F. Thus, both the lower number of antennae, as well as of galactoses in G1S-N and G1FS-N, might serve as explanations for the faster clearance compared to G2F. It is noteworthy, that the oligomannose effect was stronger than the monoantennary effect (Table S4, S10 and S11; Man5 vs G0F-N/G1FS-N p = .02 [CHO mAb1], p = .0006 [CHO mAb2], p = .0009 [ST3 mAb2]), which is in line with our previous findings.^8^ Afucosylation appeared to influence clearance in CHO mAb1 (see for example G0), but the effect direction was inconsistent between IV and SC dosing. In ST3 mAb1, effects and inconsistencies were observed as well, comparing G2S2 and G2S2F. However, while G0 was cleared faster in IV and slower in SC compared to G0F, G2S2 clearance was similar in IV and faster in SC compared to G2S2F (Table 1 and Figure 3; p = .02). In contrast CHO mAb2 did not show an afucosylation effect (Table S10), but there was a faster clearance of G2S2 compared to G2S2F in ST3 mAb2 (Table S11; p = .01). Galactosylation consistently decreased clearance in CHO mAb1 and ST3 mAb1, most prominently in SC. G1F showed slower clearance than G0F in SC with both mAbs. Clearance of G2F was even slower compared to G1F in CHO mAb1 (p < .0001). Of note, the slower clearance of G2FS2 compared to G2F in ST3 mAb2 suggested a further contribution of sialylation (Table S11), although judging by the difference in mean and the absence of a detectable difference between G2F and G2FS1, if present, it is smaller than the galactosylation effect observed in mAb1.

**Table 1.** Absolute clearance values per glycoform for the three mAb1 glycovariants in IV and SC. Importantly, absolute clearance values should not be compared between different mAb1 preparations due to the effects of glycan pairing.

Variants	CHO mAb1 CL (mL/day/kg)^1^		M5 mAb1 CL (mL/day/kg)^1^		ST3 mAb1CL (mL/day/kg)^1^	
	IV	SC	IV	SC	IV	SC
Man5	1.04 ± 0.11**	1.40 ± 0.50	1.30 ± 0.36	1.35 ± 0.28*		
G0F-N	0.95 ± 0.09**	1.71 ± 0.32				
G0	0.90 ± 0.09**	1.14 ± 0.20*				
G0F (Ref)	0.88 ± 0.09	1.54 ± 0.29	1.23 ± 0.38	1.07 ± 0.14	1.05 ± 0.18	1.85 ± 0.41
G1	0.82 ± 0.09*	1.06 ± 0.19***				
G1F	0.87 ± 0.09	1.19 ± 0.24***	1.36 ± 0.42**	1.29 ± 0.21**	0.96 ± 0.13	1.42 ± 0.33***
G2F	0.88 ± 0.10	1.02 ± 0.23****				
G2FS1					0.84 ± 0.15**	1.05 ± 0.23***
G2S2					0.79 ± 0.08	1.15 ± 0.28***
G2FS2					0.84 ± 0.10	1.08 ± 0.26***
H6N4F1S2					0.92 ± 0.11	1.08 ± 0.22**
Total	0.88 ± 0.09^2^	1.41 ± 0.27^2^	1.29 ± 0.36^2^	1.33 ± 0.27^2^	0.85 ± 0.10^2^	1.14 ± 0.27^2^

Color code for fold change in clearance compared to G0F: 0.5–0.75; 0.75–1; 1; 1–1.25. Values depicted are mean and standard deviation (n = 5). ^1^For SC, CL/F – apparent clearance as a function of bioavailability following SC administration – was used.

*p> cutoff (see Tables S4 to S11); **p > 0.01; ***p > 0.001; ****p > 0.0001;^2^not tested.

It is noteworthy, that differences between the mAb1 glycovariants cannot be detected by ELISA alone. This also previously observed limited sensitivity is likely due to the large inter-individual variation in the PK parameters which is efficiently eliminated as confounder with our glycoform-resolved approach.^8^

**Figure 4.**
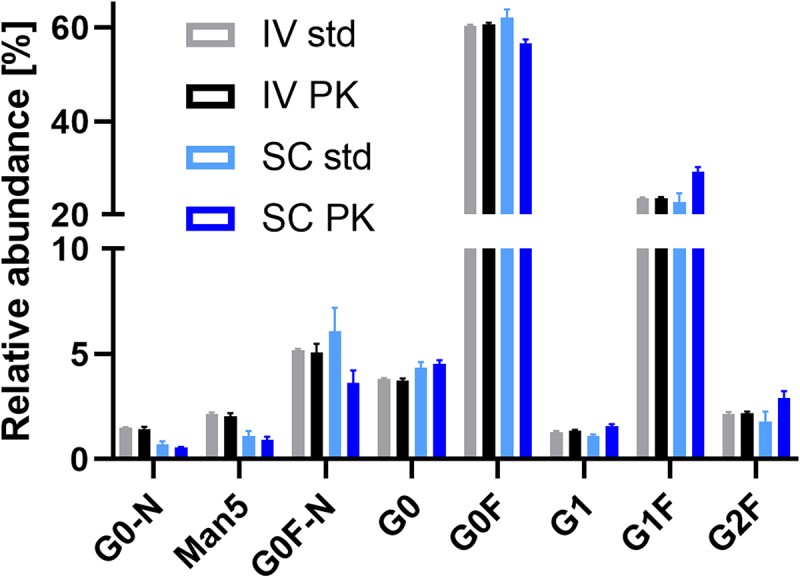
Comparison of the serum profile of CHO mAb1 after intravenous and subcutaneous injection at 24 h (c_max_ for SC; all samples and standards had concentrations of ca 5 μg/mL total mAb1). Glycoforms, where IV PK differs from SC PK, but not IV std from SC std, are indicated (*p > .01; **p > .001; ***p > .0001). Galactosylation was significantly increased in the SC profiles compared to the IV profiles. Mean and standard deviation are depicted; IV and SC std n = 3, IV and SC PK n = 5.

We observed two major differences between the glycoform-resolved PK profiles of IV and SC. Firstly, glycoform PK effects were more pronounced in SC (Table 1 and Figure 3), except for Man5 and G0F-N, which showed low precision in the SC of CHO mAb1 (Table 1 and Figure 3). Secondly, the serum profiles of the CHO mAb1 were significantly different between IV and SC (Figure 4). Note that IV profiles were largely consistent with profiles from spiked serum. Galactosylation was increased in SC (18.6%±0.9%) versus IV (15.1%±0.2%) serum profiles (p = .0007). The monoantennary glycoform G0F-N was decreased in SC (3.7%±0.6%) versus IV (5.2%±0.4%) (p = .002). Other minor effects may be attributed to analytical batch effects, as they were also observed for spiked serum samples.

## Discussion

We present herein the first glycoform-resolved PK study in minipig. The main analytical challenge is the mAb glycosylation analysis at concentrations down to 1 mg/L in the presence of a 20,000-fold excess of natural minipig antibodies.^21^ Differences in glycosylation profiles between mAbs and minipig IgG can further exaggerate this excess for low abundant glycoforms. Next to affinity enrichment, key selectivity was achieved by LC-MS with protein- and IgG subclass-specific detection of antibody glycosylation at the glycopeptide level.^22^ After mAb enrichment, minipig antibodies showed tryptic glycopeptides with masses and retention comparable to human IgG4, and thus did not interfere with the detection of IgG1 mAb-derived glycopeptides. Finally, the selected workflow also needed to provide sufficient throughput to tackle the 475 samples analyzed in this study.

Many of the observed effects were in line with previous reports for other animal models. The impact of oligomannose glycans, for example, was previously established in mice, rats, and humans.^7,8,23^ We could also confirm the effect of monoantennary glycoforms and its clear difference with the larger oligomannose effect, as previously observed in rats.^8^ Similar to the findings in rats, we did not observe a noteworthy effect of sialylation on clearance in minipigs.^8^

In contrast to the absence of a strong sialylation effect, there was an up to 50% slower clearance of galactosylated glycoforms. We could demonstrate such an effect for the first time in Fc-only glycosylated mAbs, although we observed indications of it previously.^8^ Interestingly, for M5 mAb1, galactosylation led to a faster clearance. This clear-cut difference versus CHO mAb1 and ST3 mAb1, invites the speculation that a different pathway may become impactful for M5 mAb1. Afucosylated IgG1, like M5 mAb1, are known for their stronger binding of Fc gamma receptor (FcγR) and activation of associated pathways.^24^ However, Fc gamma receptor (FcγR)-dependent TMDD usually does not contribute strongly to overall clearance and no overall difference in PK has been reported for afucosylated versus fucosylated mAbs.^25^ Nonetheless, the absence of a galactosylation effect in CHO mAb2 also suggests the potential involvement of TDMM.

The two types of injection showed a remarkable difference during the distribution phase. The glycoform distribution of the CHO mAb in the serum compartment varied markedly after SC from that after IV and the spiked serum controls (Figure 4). Galactosylation was significantly increased around *c*_max_ in SC. Either there was a strong and immediate clearance of agalactosylated glycoforms in the tissue or a prejudice in transport from tissue to serum, favoring galactosylated glycoforms. Either way, this likely means that the target tissues would have been exposed to a more galactosylated therapeutic antibody after SC than after IV. Consequently, given similar total antibody AUCs, stronger ADCC, through increased FcγR binding, and stronger complement-dependent cytotoxicity, through increased hexamerization potential for C1q binding, could be expected after SC injection.^26,27^ With an absolute increase of 3.5% in galactosylation (15.1% IV to 18.6% SC), the total impact might be limited, but not necessarily negligible.

## Materials and methods

### Glycoengineering

All mAbs were of the IgG1 subclass. CHO mAb1, M5 mAb1 and ST3 mAb1 had the same amino acid sequence. They were prepared inhouse according to a previously published protocol.^8^ In short, the CHO mAb1 was produced in CHO cells under typical conditions. For the generation of M5 mAb1, this system was supplemented with kifunensine and subsequently the purified antibody was treated with mannosidase A (in house). Finally, 10% of CHO mAb1 was added to achieve the profile shown in Figure 2b. ST3 mAb1 was obtained by consecutive chemo-enzymatic treatment of CHO mAb1 with β(1–4)-galactosyltransferase (Roche, cat. no. 08098182103) and α2,3-sialyltransferase (Roche, cat. no. 07429916103). All enzymatic steps were followed by Protein A chromatography-based purification of the product.

mAb2 Fc effector functions were silenced with the PGLALA mutation. mAb2 had a different amino acid sequence than mAb1, but CHO mAb2 and ST3 mAb2 had identical sequences. Glycoengineering of the ST3 variant of mAb2 was achieved in the same way as for mAb1.

### Single-dose pharmacokinetic study

The reported animal studies were performed in accordance with animal welfare laws and were approved by the Covance Harrogate ethical review committee. The three glycovariants of mAb1 (CHO mAb1, M5 mAb1 and ST3 mAb1) and the two glycovariants of mAb2 (CHO mAb2 and ST3 mAb2), were studied in eight independent experiments in a porcine model with five biological replicates for each experiment. mAb1 glycovariants were given IV and SC in separate experiments, while mAb2 glycovariants were only administered SC. Female Göttingen minipigs (Sus scrofa domesticus; Charles River, UK) received a single IV or SC injection of only one of the five mAb glycovariants, although each glycovariant contained a mixture of glycoforms (Figure 2 and Figure S1). Antibodies were administered as a bolus via the ear vein for IV and in the inguinal area for SC at a nominal dose of 0.5 mg/kg and 2 mg/kg for mAb1 and mAb2, respectively. These dosages present a compromise between the limited availability of the precious glycoengineerd mAbs and the ability to observe TDMM effects on the one hand, and concentration sensitivity of the LC-MS method on the other hand. The same rationale excluded a multi-dose study. Antibodies were diluted in a solution of 5 mM histidine, 60 mM trehalose, 0.01% Tween 20 pH 6.0 prior to application. All five animals in each of the eight groups were sampled at 0.08, 1, 7, 24, 48, 72, 96, and 168 h after IV injection for mAb1; at 2, 7, 24, 48, 72, 96, 168, and 240 h after SC injection for mAb1; and 2, 7, 24, 48, 72, 96, 168, 240, 336, 504, 672, 840, and 1008 h for mAb2. For the following time points no glycosylation profiles could be obtained as mAb concentrations were below the limit of quantification: CHO mAb1 IV 168 h, M5 mAb1 IV 168 h, and CHO mAb1 SC 240 h. Blood samples were taken from the jugular vein of each animal and were allowed to clot at room temperature, after which they were centrifuged at 1760 x*g* and 4°C for 10 min. Thereafter, serum samples were stored at −20°C.

### Concentration measurements

mAb concentrations in the serum samples were quantified with an ELISA method specific for the human IgG kappa chain as described before.^8^ The lower limit of quantitation was 7 ng/mL in 100% matrix (minipig serum). The assay had a dynamic range of 7 to 300.000 ng/ml in 100% matrix.

### Purification of mAbs from minipig serum and LC-MS glycopeptide analysis

As an external standard for each series of related samples, for example all IV samples of CHO mAb1, the respective mAb formulation was spiked into blank minipig serum at various concentrations: 10, 5, 1 and 0.25 μg/mL. Purification of human IgG1 mAbs from minipig serum was performed similarly to a previously reported glycoform-resolved PK analysis in rats.^8^ However, larger serum volumes were used because these were available from the larger animals and the mAb concentrations were generally lower. 100 μL of minipig serum was diluted with 150 μL phosphate-buffered saline and incubated for 1 h with 1 μL CaptureSelect™ FcXL affinity matrix (agarose beads with immobilized anti-IgG antibody; ThermoFisher Scientific). After three wash steps with 200 μL phosphate-buffered saline and then three with 200 μL water, purified mAbs were eluted by centrifugation at 450 x g for 180 s in 100 μL 100 mM formic acid (analytical grade; Sigma-Aldrich Steinheim, Germany). Samples were dried, re-dissolved in 20 μL 50 mM ammonium bicarbonate, denatured at 100°C for 5 min in 0.2% (w/v) RapiGest™ (Waters Chromatography, Etten-Leur, The Netherlands) and subjected to proteolytic cleavage with 200 ng sequencing grade trypsin (Promega, Leiden, The Netherlands) overnight. Afterward, RapiGest™ was precipitated with a final concentration of 0.6% trifluoroacetic acid. The supernatant was subjected to hydrophilic interaction chromatography – solid phase extraction as described previously, except for an increased amount of cotton and 100 μL for all volumes.^28^ Purified tryptic glycopeptides were separated by RP-nanoLC on an Acclaim PepMap 100 C18 column 150 × 0.075 mm with 3 μm particles providing a binary gradient at 700 nL/min with an Ultimate 3000 RSLCnano LC system (ThermoFisher Scientific). The starting percentage of B was lowered to 1% and re-equilibration extended (24 to 58 min) compared the previous protocol.^8^ Online MS detection occurred on a maXis™ quadrupole-time-of-flight mass spectrometer equipped with a nanoBooster™ nanoESI source (Bruker Daltonics, Bremen, Germany).^8,22^

### Data processing, non-compartmental PK analysis and statistics

Raw LC-MS data was pre-processed automatically with LaCyTools version 1.0.1 build 8.^22,29^ Parameters were the same as used previously:^8^ mass window 0.065 Th (0.08 Th for ST3 mAb1), time window 16 s (14 s for M5 mAb1 IV), background window 10, minimum isotopologue coverage 0.9, charge states 2+ and 3 + . The late PK samples and low spiked concentrations feature very low signal intensities. In these cases, next to the automatically subtracted background, specific interferences can lead to an overestimation of low abundant glycoforms. Judging by the concentration range of spiked samples, we could reasonably estimate these interferences by subtracting a fraction of 0.3 of the background – as calculated by LaCyTools – from the signals in addition. This further reduced the limited concentration dependence of the profiles.

Combining relative glycosylation profiles from LC-MS (Figures S2 and S3) and absolute total mAb concentrations, individual glycoform concentrations were calculated. This was done per animal and time point. Glycoform-resolved PK parameters were obtained from these individual concentrations by non-compartmental analysis, using the Phoenix® WinNonlin program (6.4 NY, USA) for the kinetic evaluation. For dose adjustment, individual concentrations calculated in serum spiked with 10 mg/L mAb were used (Table S12). PK parameters and glycosylation profiles were visualized and statistically treated with GraphPad Prism 9 (GraphPad Software, San Diego, US). Glycoform-resolved PK parameters were compared using paired t tests. Serum profiles were compared using an unpaired t-test with Welch correction (see Figure 4). The alpha values were adjusted per experimental group from a common 0.05 using the Benjamini–Hochberg approach with a 5% false discovery rate to correct for multiple testing. Figure 1 is created with BioRender.com.

## Supplementary Material

Supplemental Material

## Data Availability

IgG Fc N-glycan profiles and ELISA-based concentrations can be found in the supplementary information files (Table S2, S3 and S12). All analyses in this study are based on these values.
