# Supplementary material for: Clearance of therapeutic antibody glycoforms after subcutaneous and intravenous injection in a porcine model
Source: MAbs. 2022 Nov 16;14(1):2145929. doi: 10.1080/19420862.2022.2145929 (PMC9673920; doi:10.1080/19420862.2022.2145929)
Supplement: Supplemental Material [file KMAB_A_2145929_SM7228.zip › Falck et al Supplementary Information.docx]

**Clearance of therapeutic antibody glycoforms after subcutaneous and intravenous injection in a porcine model**

David Falck^1*^, Martin Lechmann^3^, Ana Momčilović^1^, Marco Thomann^2^, Carolien A. M. Koeleman^1^, Cordula Jany^3^, Sebastian Malik^3^, Manfred Wuhrer^1,$^ and Dietmar Reusch^2,$^

^1^ Center for Proteomics and Metabolomics, Leiden University Medical Center, Leiden, The Netherlands

^2^ Pharma Technical Development Europe, Roche Diagnostics GmbH, Penzberg, Germany

^3^ Roche Pharma Research and Early Development, Pharmaceutical Sciences, Roche Innovation Center Munich, Penzberg, Germany

^$^ Authors have contributed equally

*To whom correspondence should be addressed: [d.falck@lumc.nl](mailto:d.falck@lumc.nl)

Table S1: Nomenclature, categorization, compositions and structure

| Glycan name | Composition* | Glycan type | Structure** |
| --- | --- | --- | --- |
| G0F | H3N4F1 | diantennary complex |  |
| G1F | H4N4F1 | diantennary complex |  |
| G2F | H5N4F1 | diantennary complex | 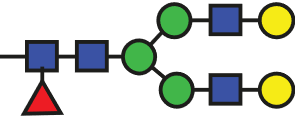 |
| Man5 | H5N2 | oligomannose |  |
| G0F-N | H3N3F1 | monoantennary complex |  |
| G0 | H3N4 | diantennary complex |  |
| G1 | H4N4 | diantennary complex | 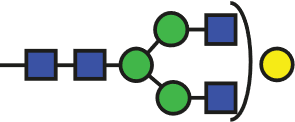 |
| G2FS1 | H5N4F1S1 | diantennary complex |  |
| G2FS2 | H5N4F1S2 | diantennary complex |  |
| G2S1 | H5N4S1 | diantennary complex | 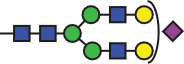 |
| G2S2 | H5N4S2 | diantennary complex |  |
| G1S1-N | H4N3S1 | monoantennary complex | 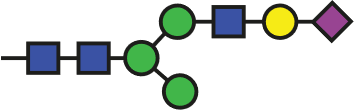 |
| G1FS1-N | H4N3F1S1 | monoantennary complex |  |
| Not determined | H6N4F1S2 | Not determined | Not determined |

* elemental composition deduced from MS measurements expressed as monosaccharide units: H = hexose, N = *N*-acetylhexosamine, F = deoxyhexose (fucose); S = sialic acid (*N*-acetylneuraminic acid)

**CFG notation


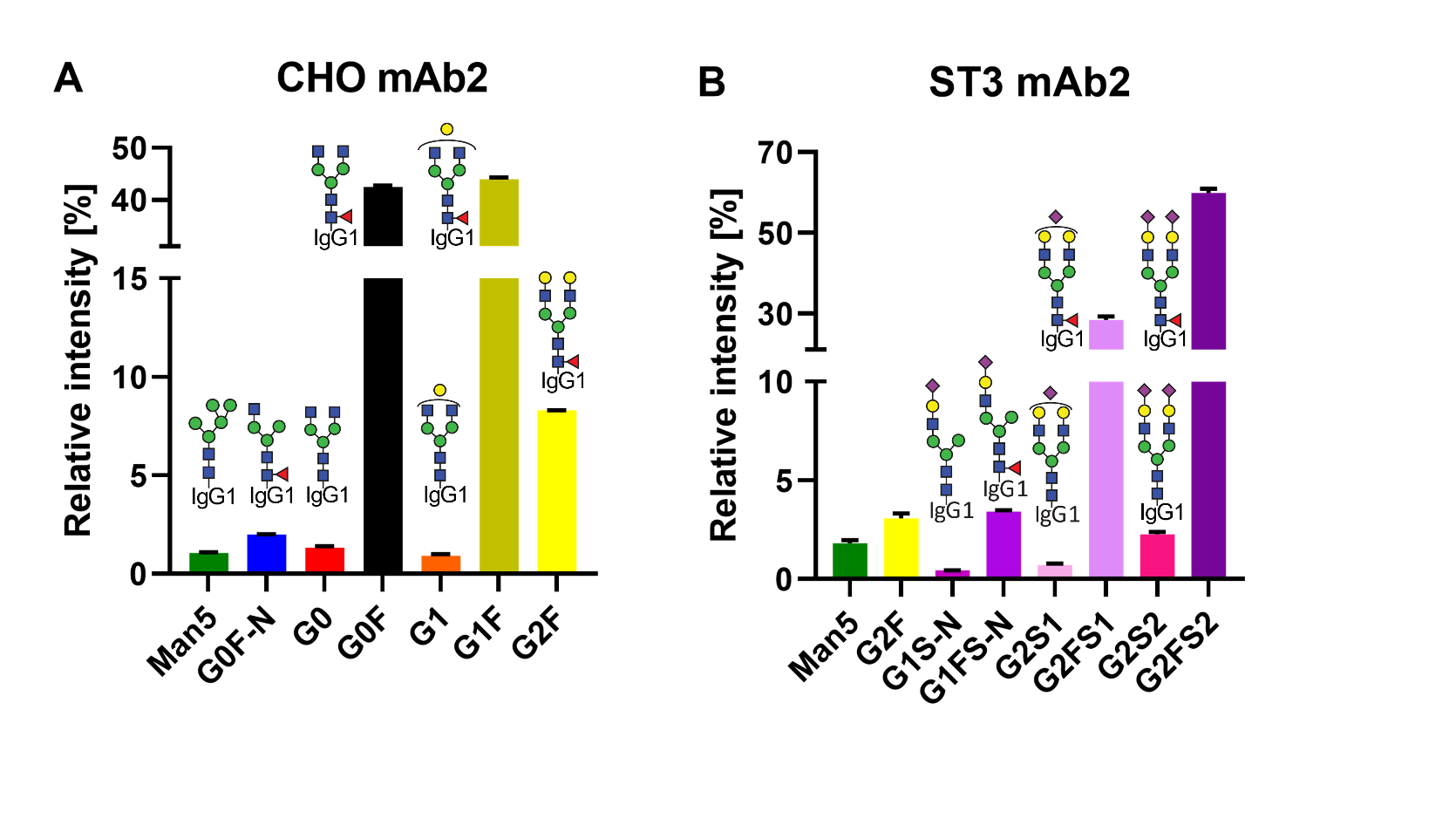


**Figure S1**: Relative abundance of quantified glycoform in the mAb2starting materials.

**Table S4:** Pharmacokinetic parameters of the different glycoforms in the **CHO mAb1** after **intravenous** application

| Name | Clearance (mL/day/kg) | Half-life (h) | AUClast/dose^3^ (h*kg*ng/mL/mg) | Relative starting content as percentage  Average ± standard deviation |
| --- | --- | --- | --- | --- |
| Man5 | 1.04±0.11** | 34.7±3.9* | 8.2·10^5^±0.8·10^5^ ** | 2.3±0.1% |
| G0F-N | 0.95±0.09** | 37.2±3.3 | 8.8·10^5^±0.7·10^5^ ** | 5.5±0.7% |
| G0 | 0.90±0.09** | 38.5±3.6 | 9.2·10^5^±0.9·10^5^ * | 3.9±0.1% |
| G0F^1^ | 0.88±0.09 | 38.2±3.7 | 9.5·10^5^±1.0·10^5^ | 60.9±0.8% |
| G1 | 0.82±0.09* | 39.8±4.6 | 9.8·10^5^±1.1·10^5^ | 1.3±0.1% |
| G1F | 0.87±0.09 | 39.2±4.1** | 9.4·10^5^±1.0·10^5^ | 23.8±>0.05% |
| G2F | 0.88±0.10 | 38.9±4.0 | 9.4·10^5^±1.1·10^5^ | 2.2±>0.05% |
| Total^2^ | 0.88±0.09 | 38.4±3.7 | 9.4·10^5^±1.0·10^5^ |  |

*p<0.025; **p<0.01;
^1^ reference;
^2^ not tested;
^3^ Area under the curve from the time of dosing to the time of the last measured concentration normalized to the dose

**Table S5:** Pharmacokinetic parameters of the different glycoforms in the **CHO mAb1** after **subcutaneous** application

| Name | Clearance (mL/day/kg) | Half-life (h) | AUClast/dose^3^ (h*kg*ng/mL/mg) | Cmax/dose^4^ (kg*ng/mL/mg) | Relative starting content as percentage  Average ± standard deviation |
| --- | --- | --- | --- | --- | --- |
| Man5 | 1.40±0.50 | 70±30 | 6.3·10^5^±3.2·10^5^ | 7.7·10^3^±3.4·10^3^ | 0.9±0.2% |
| G0F-N | 1.71±0.32 | 73±31 | 4.8·10^5^±1.5·10^5^ *** | 5.4·10^3^±1.9·10^3^ ** | 5.3±1.1% |
| G0 | 1.14±0.20* | 58±18* | 7.7·10^5^±2.1·10^5^ ** | 8.0·10^3^±2.7·10^3^ ** | 4.1±0.2% |
| G0F^1^ | 1.54±0.29 | 47±12 | 6.0·10^5^±1.6·10^5^ | 6.6·10^3^±2.3·10^3^ | 62.6±0.8% |
| G1 | 1.06±0.19*** | 54±18 | 8.5·10^5^±2.2·10^5^ *** | 9.4·10^3^±3.6·10^3^ ** | 1.2±>0.05% |
| G1F | 1.19±0.24*** | 44±9 | 8.1·10^5^±2.3·10^5^ ** | 8.9·10^3^±3.5·10^3^ * | 23.9±0.7% |
| G2F | 1.02±0.23**** | 45±7 | 9.5·10^5^±3.0·10^5^ ** | 11.1·10^3^±4.8·10^3^ * | 2.0±0.1% |
| Total^2^ | 1.41±0.27 | 48±11 | 6.6·10^5^±1.8·10^5^ | 7.2·10^3^±2.6·10^3^ |  |

*p<0.027; **p<0.01; ***p<0.001; ****p<0.0001;
^1^ reference;
^2^ not tested;
^3^ Area under the curve from the time of dosing to the time of the last measured concentration normalized to the dose;
^4^ Maximum concentration observed normalized to the dose

**Table S6:** Pharmacokinetic parameters of the different glycoforms in the **M5 mAb1** after **intravenous** application

| Name | Clearance (mL/day/kg) | Half-life (h) | AUClast/dose^3^ (h*kg*ng/mL/mg) | Relative starting content as percentage  Average ± standard deviation |
| --- | --- | --- | --- | --- |
| Man5 | 1.30±0.36 | 35.7±3.7** | 6.9·10^5^±1.6·10^5^ | 91.1±0.5% |
| G0F^1^ | 1.23±0.38 | 47.3±7.4 | 6.6·10^5^±1.6·10^5^ | 4.3±0.1% |
| G1F | 1.36±0.42** | 43.8±7.3*** | 6.2·10^5^±1.5·10^5^ ** | 4.6±0.4% |
| Total^2^ | 1.29±0.36 | 37.2±5.0 | 6.8·10^5^±1.6·10^5^ |  |

*p<0.033; **p<0.01; ***p<0.001;
^1^ reference;
^2^ not tested;
^3^ Area under the curve from the time of dosing to the time of the last measured concentration normalized to the dose

**Table S7:** Pharmacokinetic parameters of the different glycoforms in the **M5 mAb1** after **subcutaneous** application

| Name | Clearance (mL/day/kg) | Half-life (h) | AUClast/dose^3^ (h*kg*ng/mL/mg) | Cmax/dose^4^ (kg*ng/mL/mg) | Relative starting content as percentage  Average ± standard deviation |
| --- | --- | --- | --- | --- | --- |
| Man5 | 1.35±0.28* | 37.6±4.7* | 7.6·10^5^±1.6·10^5^ * | 8.8·10^3^±2.1·10^3^ | 90.5±2.8% |
| G0F^1^ | 1.07±0.14 | 49.9±8.0 | 9.0·10^5^±1.2·10^5^ | 10.7·10^3^±4.8·10^3^ | 4.2±0.7% |
| G1F | 1.29±0.21** | 44.5±5.0 | 7.7·10^5^±1.3·10^5^ ** | 8.4·10^3^±2.3·10^3^ | 5.3±2.1% |
| Total^2^ | 1.33±0.27 | 38.8±4.7 | 7.7·10^5^±1.5·10^5^ | 8.8·10^3^±2.0·10^3^ |  |

*p<0.020; **p<0.01;
^1^ reference;
^2^ not tested;
^3^ Area under the curve from the time of dosing to the time of the last measured concentration normalized to the dose;
^4^ Maximum concentration observed normalized to the dose

**Table S8:** Pharmacokinetic parameters of the different glycoforms in the **ST3 mAb1** after **intravenous** application

| Name | Clearance (mL/day/kg) | Half-life (h) | AUClast/dose^3^ (h*kg*ng/mL/mg) | Relative starting content as percentage  Average ± standard deviation |
| --- | --- | --- | --- | --- |
| G0F^1^ | 1.05±0.18 | 35.5±8.0 | 9.4*10^5^±1.8*10^5^ | 6.4±0.2% |
| G1F | 0.96±0.13 | 42.7±24.4 | 9.7*10^5^±1.0*10^5^ | 2.6±0.3% |
| G2FS1 | 0.84±0.15** | 46.2±2.8 | 11.5*10^5^±2.4*10^5^ ** | 4.7±0.2% |
| G2S2 | 0.79±0.08 | 45.8±2.6 | 11.9*10^5^±1.1*10^5^ | 5.1±0.2% |
| G2FS2 | 0.84±0.10 | 47.9±2.4 | 11.0*10^5^±1.3*10^5^ | 80.6±0.5% |
| H6N4F1S2 | 0.92±0.11 | 42.7±3.0 | 10.3*10^5^±1.1*10^5^ | 0.7±>0.05% |
| Total^2^ | 0.85±0.10 | 46.8±2.6 | 10.9*10^5^±1.2*10^5^ |  |

**p<0.0067;
^1^ reference;
^2^ not tested;
^3^ Area under the curve from the time of dosing to the time of the last measured concentration normalized to the dose

**Table S9:** Pharmacokinetic parameters of the different glycoforms in the **ST3 mAb1** after **subcutaneous** application

| Name | Clearance (mL/day/kg) | Half-life (h) | AUClast/dose^3^ (h*kg*ng/mL/mg) | Cmax/dose^4^ (kg*ng/mL/mg) | Relative starting content as percentage  Average ± standard deviation |
| --- | --- | --- | --- | --- | --- |
| G0F^1^ | 1.85±0.41 | 43±6 | 5.5*10^5^±1.3*10^5^ | 6.2*10^3^±1.8*10^3^ | 6.9±0.4% |
| G1F | 1.42±0.33*** | 40±13 | 7.2*10^5^±1.9*10^5^ ** | 8.5*10^3^±2.5*10^3^ ** | 2.7±0.2% |
| G2FS1 | 1.05±0.23*** | 64±8*** | 8.9*10^5^±2.1*10^5^ ** | 7.1*10^3^±1.6*10^3^ * | 6.1±0.4% |
| G2S2 | 1.15±0.28*** | 56±7** | 8.5*10^5^±2.1*10^5^ ** | 10.0*10^3^±3.0*10^3^ * | 4.7±0.1% |
| G2FS2 | 1.08±0.26*** | 59±7** | 8.9*10^5^±2.2*10^5^ ** | 9.2*10^3^±2.5*10^3^ ** | 78.9±0.9% |
| H6N4F1S2 | 1.08±0.22** | 70±23 | 8.5*10^5^±2.3*10^5^ ** | 9.6*10^3^±2.6*10^3^ ** | 0.7±>0.05% |
| Total^2^ | 1.14±0.27 | 59±7 | 8.6*10^5^±2.0*10^5^ | 8.8*10^3^±2.3*10^3^ |  |

*p<0.039; **p<0.01; ***p<0.001;
^1^ reference;
^2^ not tested;
^3^ Area under the curve from the time of dosing to the time of the last measured concentration normalized to the dose;
^4^ Maximum concentration observed normalized to the dose

**Table S10:** Pharmacokinetic parameters of the different glycoforms in the **CHO mAb2** after **subcutaneous** application

| Name | Clearance (mL/day/kg) | Half-life (h) | AUClast/dose^3^ (h*kg*ng/mL/mg) | Cmax/dose^4^ (kg*ng/mL/mg) | Relative starting content as percentage  Average ± standard deviation |
| --- | --- | --- | --- | --- | --- |
| Man5 | 0.32±0.05*** | 209±63 | 3.1*10^6^±0.6*10^6^ ** | 8.3*10^3^±1.1*10^3^ | 1.1±>0.05% |
| G0F-N | 0.20±0.06 | 298±120 | 4.7*10^6^±1.2*10^6^ | 9.6*10^3^±0.8*10^3^ | 2.0±>0.05% |
| G0 | 0.19±0.06 | 309±130 | 4.8*10^6^±1.2*10^6^ | 10.0*10^3^±0.9*10^3^ | 1.3±0.1% |
| G0F^1^ | 0.19±0.06 | 323±127 | 4.8*10^6^±1.3*10^6^ | 9.6*10^3^±0.7*10^3^ | 42.5±>0.2% |
| G1 | 0.19±0.06 | 289±122 | 5.0*10^6^±1.2*10^6^ *** | 11.4*10^3^±0.5*10^3^ | 0.9±>0.1% |
| G1F | 0.19±0.06 | 325±129 | 4.8*10^6^±1.3*10^6^ | 9.5*10^3^±0.7*10^3^ | 43.9±>0.4% |
| G2F | 0.19±0.06 | 313±133 | 4.8*10^6^±1.3*10^6^ | 9.9*10^3^±0.8*10^3^ ** | 8.3±>0.05% |
| Total^2^ | 0.20±0.07 | 304±150 | 4.8*10^6^±1.2*10^6^ | 9.6*10^3^±0.7*10^3^ |  |

**p<0.010; ***p<0.001; ****p<0.0001;
^1^ reference;
^2^ not tested;
^3^ Area under the curve from the time of dosing to the time of the last measured concentration normalized to the dose;
^4^ Maximum concentration observed normalized to the dose

**Table 11** Pharmacokinetic parameters of the different glycoforms in the **ST3 mAb2** after **subcutaneous** application

| Name | Clearance (mL/day/kg) | Half-life (h) | AUClast/dose^3^ (h*kg*ng/mL/mg) | Cmax/dose^4^ (kg*ng/mL/mg) | Relative starting content as percentage  Average ± standard deviation |
| --- | --- | --- | --- | --- | --- |
| Man5 | 0.52±0.16** | 402±125* | 1.6*10^6^±0.4*10^6^ *** | 3.7*10^3^±0.6*10^3^ **** | 1.8±0.2% |
| G2F^1^ | 0.14±0.06 | 548±192 | 5.6*10^6^±1.3*10^6^ | 9.8*10^3^±0.5*10^3^ | 3.1±0.3% |
| G1S-N | 0.23±0.09** | 540±188 | 3.3*10^6^±0.8*10^6^ *** | 6.0*10^3^±0.6*10^3^ **** | 0.4±>0.05% |
| G1FS-N | 0.21±0.10** | 592±209 | 3.5*10^6^±0.8*10^6^ *** | 5.8*10^3^±0.4*10^3^ **** | 3.4±0.1% |
| G2S1 | 0.11±0.05* | 752±255** | 5.5*10^6^±1.3*10^6^ | 8.9*10^3^±0.4*10^3^ ** | 0.7±0.1% |
| G2FS1 | 0.12±0.05 | 587±194 | 6.0*10^6^±1.4*10^6^ ** | 10.2*10^3^±0.5*10^3^ *** | 28.4±0.9% |
| G2S2 | 0.19±0.09* | 642±213* | 3.7*10^6^±0.9*10^6^ *** | 6.2*10^3^±0.6*10^3^ **** | 2.3±0.1% |
| G2FS2 | 0.11±0.05* | 651±234 | 6.4*10^6^±1.5*10^6^ ** | 10.5*10^3^±0.7*10^3^ ** | 59.9±1.1% |
| Total^2^ | 0.13±0.07 | 594±287 | 6.1*10^5^±1.3*10^5^ | 10.0*10^3^±0.6*10^3^ |  |

*p<0.031; **p<0.01; ***p<0.001; ****p<0.0001;
^1^ reference;
^2^ not tested;
^3^ Area under the curve from the time of dosing to the time of the last measured concentration normalized to the dose;
^4^ Maximum concentration observed normalized to the dose


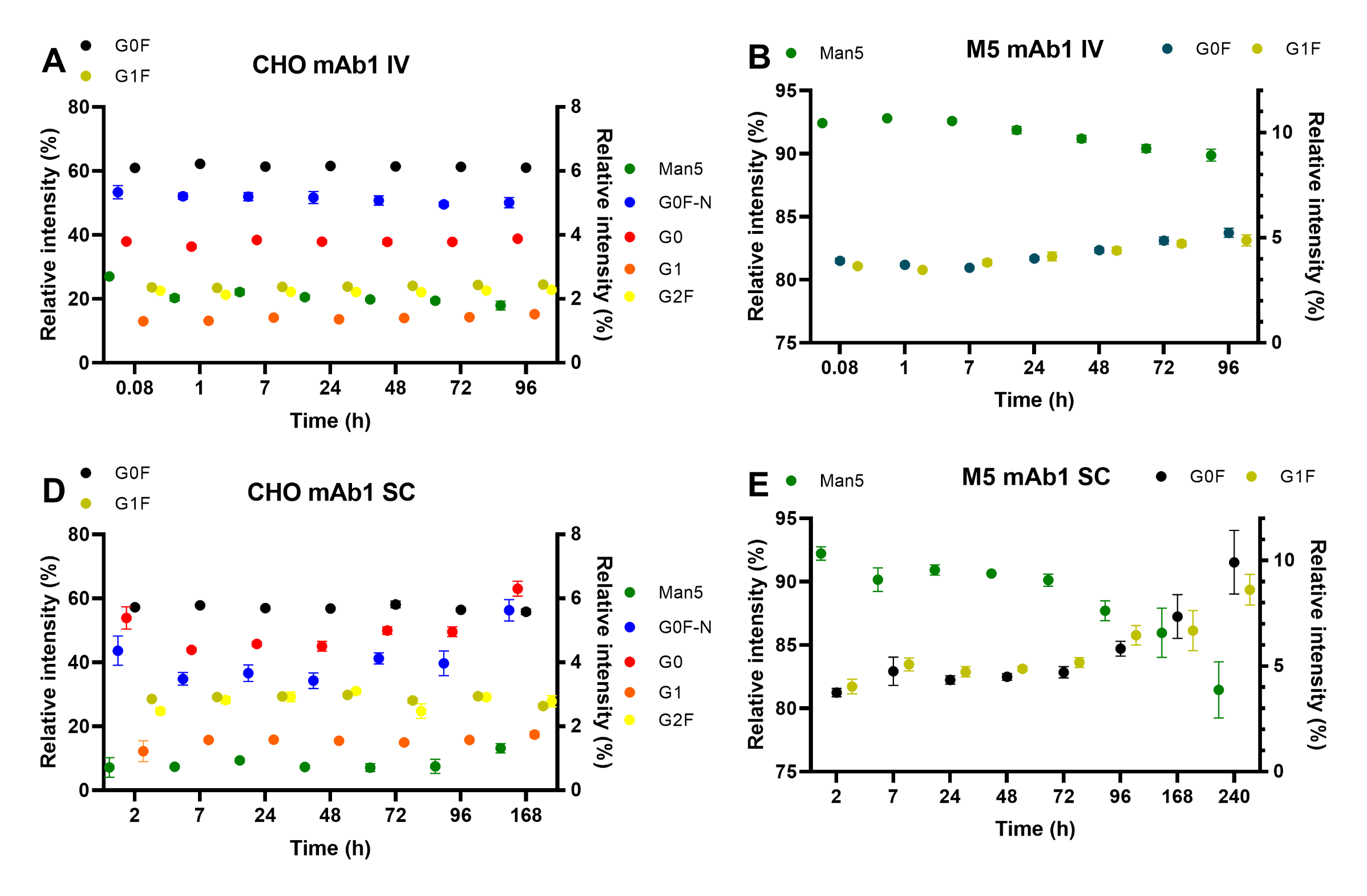


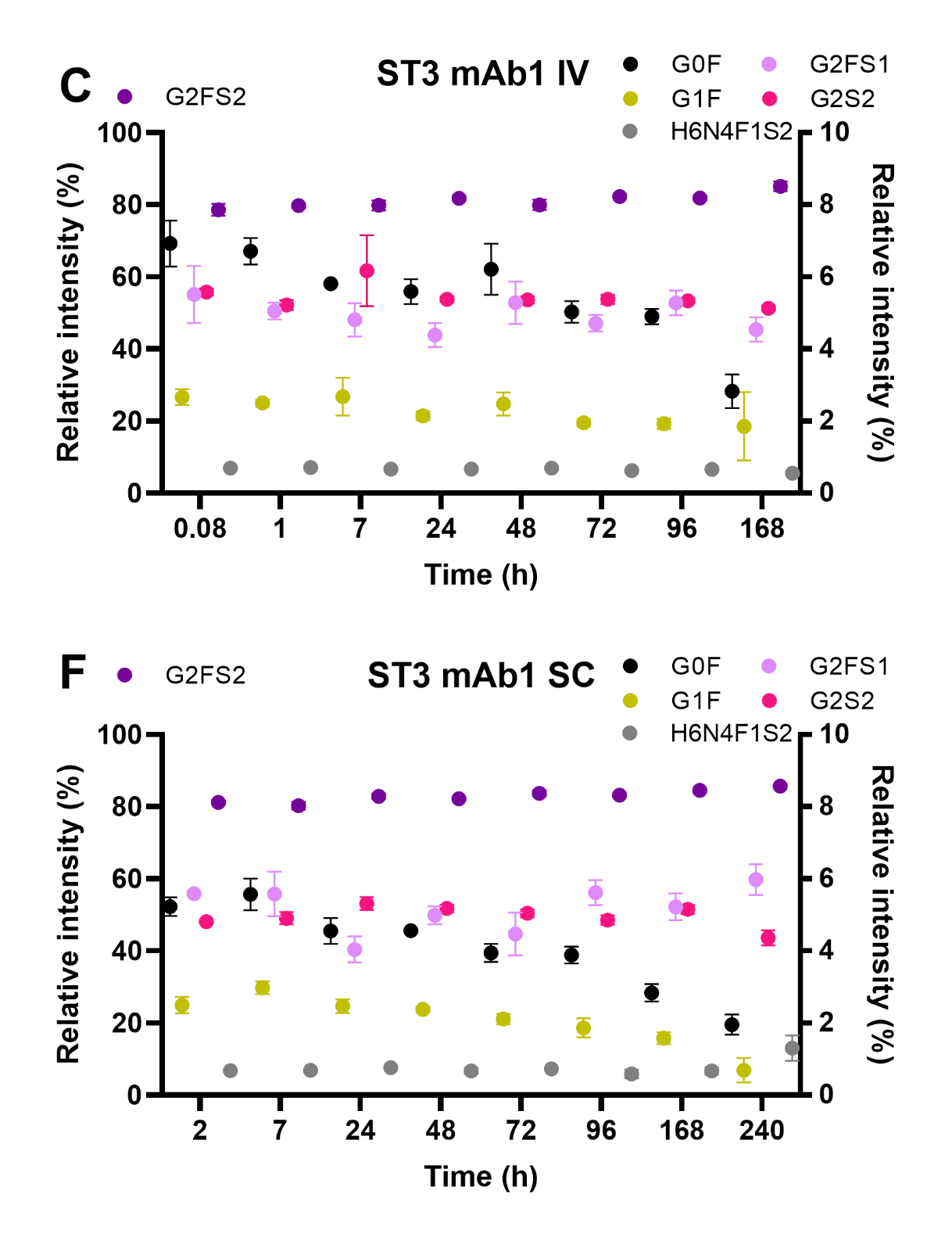


**Figure S2**: Glycosylation changes over time for mAb1 variants: A+D) CHO mAb1; B+E) M5 mAb1; C+F) ST3 mAb1. A-C) intravenous and D-F) subcutaneous injection. Error bars show mean and standard error of the mean (SEM).

**
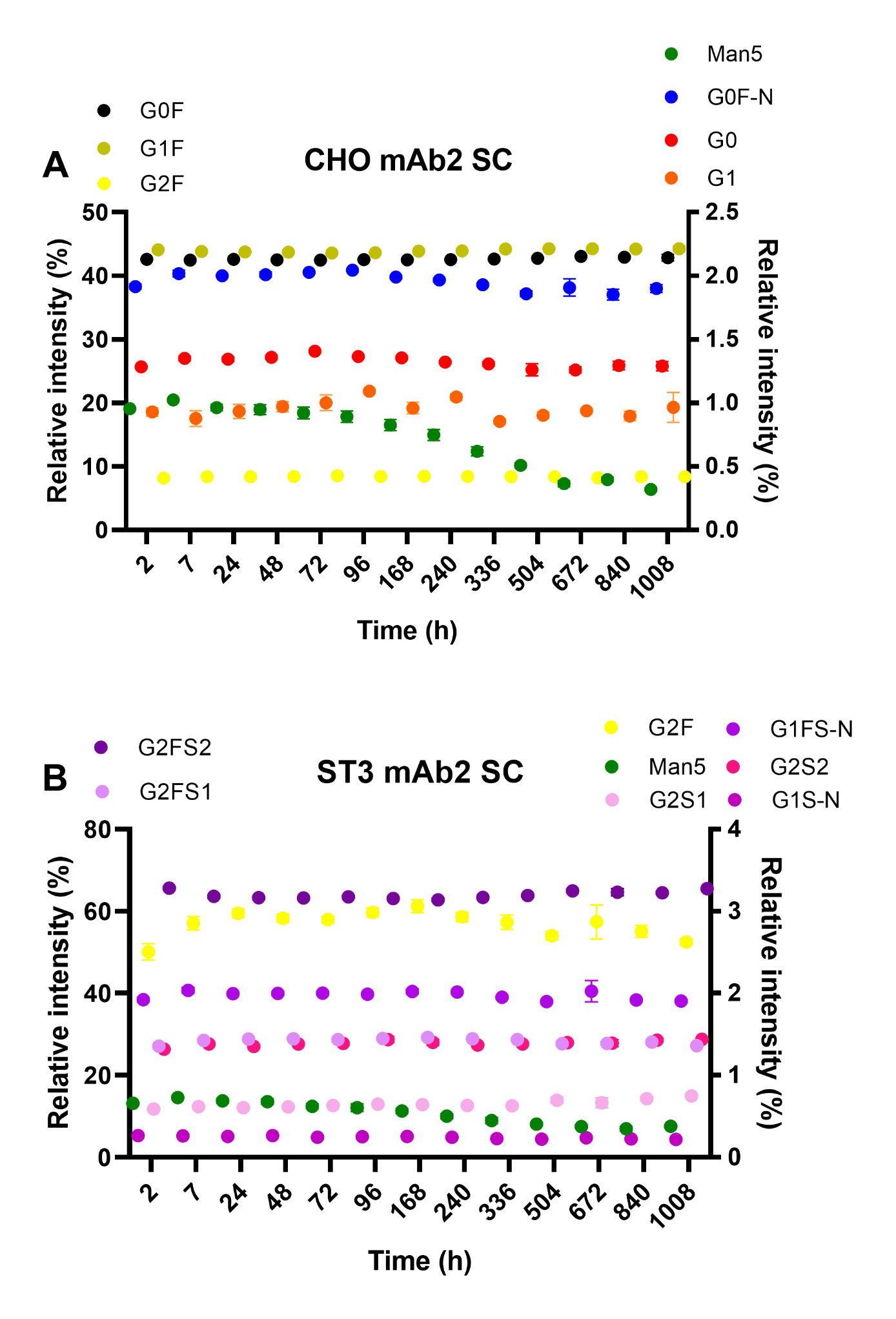
**

**Figure S3**: Glycosylation changes over time for mAb2 variants after subcutaneous injection: A) CHO mAb2; B) ST3 mAb2. Error bars show mean and standard error of the mean (SEM).

# Methods for mAb2

### Purification of mAbs from minipig serum and LC-MS glycopeptide analysis

Human IgG1 mAbs enrichment from minipig serum was performed according to previously published analysis with the following modifications.^[[1]](#footnote-1)^ 10 µL of minipig serum were diluted with 140 µL of phosphate-buffered saline (PBS) and 1 µL of CaptureSelect IgG1 (Hu) beads (8 mg/ml binding capacity; ThermoFisher Scientific), followed with the agitated incubation. Decreased amount of minipig serum prevented filter-plate clogging, thus increasing robustness of sample preparation. Reduced total capacity of immunoaffinity beads (maximum 8 µg of IgG1 Hu) favored specific mAbs enrichment despite the interference of abundant minipig immunoglobulins. ^[[2]](#footnote-2)^ Washing was performed with PBS and water, 200 µL three times each using a vacuum manifold (50 kPa pressure gradient). mAbs were eluted with 100 µL 100 mM formic acid (LC-MS grade; Sigma-Aldrich, Steinheim, Germany) at 440 x*g* for 3min, and dried by vacuum centrifugation for 2.5 h at 60 °C. Samples were re-dissolved directly in 20 µL of 0.2% (w/v) Rapigest (Waters Chromatography, Etten-Leur, The Netherlands) in 50mM ammonium bicarbonate, denatured at 60°C for 30 min according to manufacturer recommendations.^[[3]](#footnote-3)^ Proteolytic cleavage was performed overnight at 37 °C with 200 ng sequencing grade modified trypsin from Promega (Madison, WI). 1 µL of 25% trifluoroacetic acid (TFA) from Merck (Darmstadt, Germany) was used to precipitate RapiGest during 30 min incubation at 37°C. After centrifugation, 10 min at 3100 x*g*, 20 µL of supernatant with digested glycoproteins was transferred to new plate. From each sample 1 μL were analyzed in ascending concertation order by RP-nanoLC on an Acclaim PepMap 100 C18 column 150 × 0.075 mm with 3 μm particles providing a binary gradient at 600 nL/min with an Ultimate 3000 RSLCnano LC system (ThermoFisher Scientific). A linear gradient of solvent A (0.1% TFA in water) and solvent B (95% acetonitrile) was applied: 3% B 0 min, 25% B 6 min, 50% B 7 min, 50% B 9 min, 3% B 10−12.5 min. Online MS detection occurred on an Impact quadrupole-time-of-flight mass spectrometer equipped with a nanoBooster™ nanoESI source (Bruker Daltonics, Bremen, Germany), enriching the nitrogen dry gas (0.2 bar, 180 °C) with acetonitrile. ^1,^^[[4]](#footnote-4)^

### Data processing, non-compartmental PK analysis and statistics

mAb2 data was handled mostly like reported for mAb1. Setting for the LC-MS data pre-processing by LaCyTools deviated due to the use of a different mass spectrometer for data acquisition: mass window 0.1 Th for CHO mAb2 and 0.15 Th for ST3 mAb2, time window 9 s for CHO mAb2 and 10 s for ST3 mAb2, and minimum isotopologue coverage 0.85.

1. # References:

   Falck D, Thomann M, Lechmann M, Koeleman CAM, Malik S, Jany C, Wuhrer M, Reusch D. Glycoform-resolved pharmacokinetic studies in a rat model employing glycoengineered variants of a therapeutic monoclonal antibody. MAbs. 2021; 13 (1): 1865596. [↑](#footnote-ref-1)
2. https://www.thermofisher.com/order/catalog/product/191303005 [↑](#footnote-ref-2)
3. https://www.waters.com/webassets/cms/support/docs/715000122en.pdf [↑](#footnote-ref-3)
4. Falck D, Jansen BC, de Haan N, Wuhrer M. High-Throughput Analysis of IgG Fc Glycopeptides by LC-MS. Methods Mol Biol. 2017; 1503: 31-47. [↑](#footnote-ref-4)
