# Supplementary material for: Clearance of therapeutic antibody glycoforms after subcutaneous and intravenous injection in a porcine model
Source: MAbs. 2022 Nov 16;14(1):2145929. doi: 10.1080/19420862.2022.2145929 (PMC9673920; doi:10.1080/19420862.2022.2145929)
Supplement: Supplemental Material [file KMAB_A_2145929_SM7228.zip › Tables S2 S3 and S12.docx]

**Table S2A:** Glycosylation profiles for the PK study after **intravenous injection** of **CHO mAb1.**Relative intensities for the quantified glycoforms are given in percentage separately for each time point and each animal (five biological replicates).

| Time point_animal | Man5 | G0F-N | G0 | G0F | G1 | G1F | G2F |
| --- | --- | --- | --- | --- | --- | --- | --- |
| 4d_1 | 2.06% | 5.35% | 3.99% | 60.54% | 1.58% | 24.27% | 2.22% |
| 4d_2 | 1.65% | 4.77% | 3.83% | 61.36% | 1.58% | 24.57% | 2.25% |
| 4d_3 | 2.18% | 5.42% | 3.89% | 60.37% | 1.37% | 24.44% | 2.33% |
| 4d_4 | 1.48% | 4.88% | 3.86% | 61.57% | 1.53% | 24.40% | 2.28% |
| 4d_5 | 1.61% | 4.62% | 3.83% | 61.19% | 1.54% | 24.90% | 2.32% |
| 3d_1 | 1.74% | 4.73% | 3.65% | 61.80% | 1.45% | 24.34% | 2.29% |
| 3d_2 | 2.10% | 5.08% | 3.90% | 60.74% | 1.55% | 24.30% | 2.34% |
| 3d_3 | 2.04% | 4.85% | 3.84% | 61.33% | 1.33% | 24.43% | 2.19% |
| 3d_4 | 1.85% | 4.97% | 3.66% | 61.42% | 1.40% | 24.44% | 2.26% |
| 3d_5 | 1.98% | 5.16% | 3.86% | 61.05% | 1.43% | 24.29% | 2.23% |
| 2d_1 | 1.87% | 4.88% | 3.63% | 61.60% | 1.41% | 24.37% | 2.25% |
| 2d_2 | 2.13% | 5.00% | 3.80% | 61.18% | 1.48% | 24.14% | 2.28% |
| 2d_3 | 1.90% | 4.73% | 3.60% | 61.86% | 1.41% | 24.23% | 2.26% |
| 2d_4 | 2.05% | 5.58% | 3.98% | 61.05% | 1.35% | 23.83% | 2.15% |
| 2d_5 | 1.95% | 5.18% | 3.90% | 61.63% | 1.37% | 23.87% | 2.10% |
| 24h_1 | 2.34% | 5.78% | 3.90% | 61.07% | 1.27% | 23.52% | 2.13% |
| 24h_2 | 1.95% | 4.97% | 3.75% | 61.90% | 1.38% | 23.83% | 2.21% |
| 24h_3 | 1.94% | 4.97% | 3.61% | 61.76% | 1.38% | 24.08% | 2.26% |
| 24h_4 | 2.04% | 4.75% | 3.80% | 61.60% | 1.37% | 24.13% | 2.31% |
| 24h_5 | 2.02% | 5.38% | 3.90% | 61.41% | 1.42% | 23.71% | 2.17% |
| 7h_1 | 2.22% | 5.32% | 3.84% | 61.47% | 1.33% | 23.61% | 2.19% |
| 7h_2 | 2.32% | 5.47% | 3.91% | 61.15% | 1.55% | 23.41% | 2.20% |
| 7h_3 | 2.34% | 5.00% | 3.84% | 60.97% | 1.32% | 24.21% | 2.31% |
| 7h_4 | 1.85% | 4.81% | 3.78% | 62.10% | 1.49% | 23.77% | 2.20% |
| 7h_5 | 2.35% | 5.39% | 3.85% | 61.01% | 1.38% | 23.78% | 2.23% |
| 1h_1 | 2.20% | 5.22% | 3.80% | 61.74% | 1.34% | 23.54% | 2.17% |
| 1h_2 | 2.01% | 5.08% | 3.74% | 62.58% | 1.28% | 23.21% | 2.09% |
| 1h_3 | 2.29% | 5.56% | 3.67% | 61.79% | 1.32% | 23.25% | 2.11% |
| 1h_4 | 1.85% | 5.23% | 3.46% | 62.16% | 1.35% | 23.78% | 2.17% |
| 1h_5 | 1.81% | 4.96% | 3.51% | 62.84% | 1.29% | 23.47% | 2.10% |
| 5min_1 | 2.59% | 5.72% | 3.88% | 61.03% | 1.28% | 23.34% | 2.17% |
| 5min_2 | 2.74% | 4.97% | 3.90% | 61.26% | 1.29% | 23.58% | 2.26% |
| 5min_3 | 2.70% | 4.72% | 3.82% | 61.32% | 1.35% | 23.76% | 2.34% |
| 5min_4 | 2.71% | 5.63% | 3.74% | 60.75% | 1.31% | 23.64% | 2.22% |
| 5min_5 | 2.76% | 5.64% | 3.66% | 60.51% | 1.30% | 23.84% | 2.29% |

**Table S2B:** Glycosylation profiles for the PK study after **subcutaneous injection** of **CHO mAb1**Relative intensities for the quantified glycoforms are given in percentage separately for each time point and each animal (five biological replicates).

| Time point_animal | Man5 | G0F-N | G0 | G0F | G1 | G1F | G2F |
| --- | --- | --- | --- | --- | --- | --- | --- |
| 7d_1 | 1.66% | 6.33% | 6.82% | 55.51% | 2.03% | 25.00% | 2.65% |
| 7d_2 | 0.84% | 5.91% | 5.96% | 57.20% | 1.39% | 26.17% | 2.53% |
| 7d_3 | 1.34% | 4.97% | 5.70% | 58.27% | 1.76% | 25.53% | 2.42% |
| 7d_4 | 1.19% | 6.24% | 6.87% | 54.99% | 1.74% | 26.05% | 2.91% |
| 7d_5 | 1.54% | 4.70% | 6.17% | 53.23% | 1.81% | 29.15% | 3.40% |
| 4d_1 | 0.60% | 3.60% | 4.71% | 56.99% | 1.60% | 29.48% | 3.02% |
| 4d_2 | 0.61% | 3.20% | 4.58% | 56.76% | 1.62% | 30.11% | 3.12% |
| 4d_3 | 0.62% | 4.04% | 4.90% | 56.92% | 1.53% | 29.06% | 2.94% |
| 4d_4 | 0.33% | 3.60% | 5.22% | 57.17% | 1.64% | 29.49% | 2.55% |
| 4d_5 | 1.59% | 5.42% | 5.38% | 54.28% | 1.48% | 28.90% | 2.95% |
| 3d_1 | 0.29% | 4.07% | 5.36% | 60.36% | 1.43% | 26.64% | 1.86% |
| 3d_2 | 0.76% | 3.80% | 4.97% | 60.81% | 1.66% | 25.97% | 2.03% |
| 3d_3 | 0.79% | 4.67% | 4.89% | 56.85% | 1.28% | 28.81% | 2.72% |
| 3d_4 | 0.70% | 4.35% | 5.11% | 56.56% | 1.57% | 28.98% | 2.74% |
| 3d_5 | 1.01% | 3.73% | 4.64% | 56.06% | 1.55% | 29.97% | 3.04% |
| 2d_1 | 0.80% | 3.31% | 4.71% | 56.08% | 1.54% | 30.34% | 3.22% |
| 2d_2 | 0.69% | 3.11% | 4.17% | 57.02% | 1.67% | 30.10% | 3.24% |
| 2d_3 | 0.73% | 2.88% | 4.10% | 57.41% | 1.57% | 30.11% | 3.20% |
| 2d_4 | 0.57% | 3.55% | 4.81% | 56.88% | 1.49% | 29.72% | 2.98% |
| 2d_5 | 0.87% | 4.29% | 4.73% | 56.93% | 1.49% | 28.83% | 2.86% |
| 24h_1 | 0.87% | 4.02% | 4.72% | 57.85% | 1.58% | 28.36% | 2.60% |
| 24h_2 | 0.78% | 4.00% | 4.50% | 57.75% | 1.43% | 28.89% | 2.64% |
| 24h_3 | 1.10% | 3.14% | 4.57% | 56.22% | 1.59% | 30.20% | 3.18% |
| 24h_4 | 0.88% | 4.22% | 4.75% | 57.01% | 1.67% | 28.65% | 2.82% |
| 24h_5 | 1.05% | 2.94% | 4.35% | 56.06% | 1.64% | 30.59% | 3.37% |
| 7h_1 | 0.98% | 3.49% | 4.32% | 57.05% | 1.67% | 29.50% | 2.99% |
| 7h_2 | 0.74% | 3.04% | 4.24% | 57.56% | 1.64% | 29.70% | 3.07% |
| 7h_3 | 0.46% | 3.05% | 4.35% | 58.00% | 1.57% | 29.67% | 2.90% |
| 7h_4 | 0.90% | 3.86% | 4.52% | 58.34% | 1.66% | 28.09% | 2.63% |
| 7h_5 | 0.60% | 3.99% | 4.53% | 58.06% | 1.35% | 28.94% | 2.53% |
| 2h_1 | 1.15% | 3.87% | 5.50% | 57.02% | 1.65% | 28.46% | 2.34% |
| 2h_2 | 0.00% | 5.73% | 6.33% | 57.25% | 0.29% | 28.10% | 2.31% |
| 2h_3 |  |  |  |  |  |  |  |
| 2h_4 | 0.42% | 4.02% | 4.86% | 57.96% | 1.24% | 29.01% | 2.49% |
| 2h_5 | 1.29% | 3.84% | 4.88% | 56.78% | 1.71% | 28.73% | 2.77% |

**Table S2C:** Glycosylation profiles for the PK study after **intravenous injection** of **M5 mAb1**Relative intensities for the quantified glycoforms are given in percentage separately for each time point and each animal (five biological replicates).

| Time point_animal | Man5 | G0F | G1F |
| --- | --- | --- | --- |
| 4d_1 | 89.12% | 5.61% | 5.27% |
| 4d_2 | 88.77% | 5.68% | 5.55% |
| 4d_3 | 89.57% | 5.46% | 4.97% |
| 4d_4 | 91.31% | 4.64% | 4.05% |
| 4d_5 | 90.64% | 4.80% | 4.57% |
| 3d_1 | 90.40% | 4.86% | 4.74% |
| 3d_2 | 89.38% | 5.38% | 5.24% |
| 3d_3 | 90.30% | 5.07% | 4.64% |
| 3d_4 | 91.20% | 4.39% | 4.40% |
| 3d_5 | 90.79% | 4.63% | 4.58% |
| 2d_1 | 90.70% | 4.64% | 4.66% |
| 2d_2 | 90.65% | 4.65% | 4.70% |
| 2d_3 | 90.98% | 4.55% | 4.47% |
| 2d_4 | 91.53% | 4.17% | 4.30% |
| 2d_5 | 92.08% | 4.06% | 3.86% |
| 24h_1 | 91.50% | 4.05% | 4.45% |
| 24h_2 | 91.87% | 4.24% | 3.89% |
| 24h_3 | 91.24% | 4.17% | 4.59% |
| 24h_4 | 92.89% | 3.70% | 3.42% |
| 24h_5 | 91.86% | 3.92% | 4.22% |
| 7h_1 | 92.86% | 3.57% | 3.57% |
| 7h_2 | 92.21% | 3.50% | 4.29% |
| 7h_3 | 92.96% | 3.52% | 3.52% |
| 7h_4 | 92.42% | 3.64% | 3.94% |
| 7h_5 | 92.55% | 3.67% | 3.79% |
| 1h_1 | 92.71% | 3.75% | 3.54% |
| 1h_2 | 93.07% | 3.53% | 3.41% |
| 1h_3 | 92.53% | 3.89% | 3.58% |
| 1h_4 | 92.96% | 3.65% | 3.39% |
| 1h_5 | 92.80% | 3.76% | 3.44% |
| 5min_1 | 92.85% | 3.73% | 3.42% |
| 5min_2 | 92.40% | 3.95% | 3.65% |
| 5min_3 | 91.74% | 4.26% | 4.00% |
| 5min_4 | 92.90% | 3.61% | 3.49% |
| 5min_5 | 92.27% | 4.00% | 3.73% |

**Table S2D:** Glycosylation profiles for the PK study after **subcutaneous injection** of **M5 mAb1**Relative intensities for the quantified glycoforms are given in percentage separately for each time point and each animal (five biological replicates).

| Time point_animal | Man5 | G0F | G1F |
| --- | --- | --- | --- |
| 10d_1 | 83.94% | 8.12% | 7.94% |
| 10d_2 | 73.12% | 15.61% | 11.27% |
| 10d_3 | 83.95% | 8.04% | 8.01% |
| 10d_4 | 85.51% | 7.48% | 7.00% |
| 10d_5 | 80.78% | 10.35% | 8.87% |
| 7d_1 | 88.52% | 6.22% | 5.26% |
| 7d_2 | 82.15% | 9.92% | 7.93% |
| 7d_3 | 88.76% | 5.87% | 5.36% |
| 7d_4 | 89.97% | 4.99% | 5.04% |
| 7d_5 | 80.43% | 9.74% | 9.84% |
| 4d_1 | 85.22% | 6.70% | 8.08% |
| 4d_2 | 86.98% | 6.18% | 6.84% |
| 4d_3 | 88.72% | 5.45% | 5.83% |
| 4d_4 | 89.82% | 4.67% | 5.51% |
| 4d_5 | 87.81% | 6.11% | 6.08% |
| 3d_1 | 90.94% | 4.54% | 4.52% |
| 3d_2 | 88.48% | 5.56% | 5.96% |
| 3d_3 | 90.53% | 4.43% | 5.04% |
| 3d_4 | 90.99% | 4.01% | 5.00% |
| 3d_5 | 89.67% | 4.99% | 5.34% |
| 2d_1 | 90.60% | 4.40% | 5.00% |
| 2d_2 | 90.58% | 5.00% | 4.43% |
| 2d_3 | 90.27% | 4.63% | 5.11% |
| 2d_4 | 91.10% | 4.08% | 4.82% |
| 2d_5 | 90.64% | 4.35% | 5.01% |
| 24h_1 | 91.31% | 4.03% | 4.65% |
| 24h_2 | 89.33% | 5.08% | 5.59% |
| 24h_3 | 91.49% | 4.46% | 4.05% |
| 24h_4 | 91.18% | 4.13% | 4.70% |
| 24h_5 | 91.31% | 4.03% | 4.66% |
| 7h_1 | 91.89% | 3.71% | 4.40% |
| 7h_2 | 90.62% | 4.54% | 4.84% |
| 7h_3 | 89.50% | 4.68% | 5.82% |
| 7h_4 | 91.91% | 3.55% | 4.54% |
| 7h_5 | 86.89% | 7.30% | 5.81% |
| 2h_1 | 93.32% | 3.44% | 3.24% |
| 2h_2 | 91.17% | 4.17% | 4.66% |
| 2h_3 | 90.87% | 4.29% | 4.84% |
| 2h_4 | 92.31% | 3.46% | 4.23% |
| 2h_5 | 93.44% | 3.37% | 3.19% |

**Table S2E:** Glycosylation profiles for the PK study after **intravenous injection** of **ST3 mAb1**Relative intensities for the quantified glycoforms are given in percentage separately for each time point and each animal (five biological replicates).

| Time point_animal | G0F | G1F | G2FS1 | G2S2 | G2FS2 | H6N4F1S2 |
| --- | --- | --- | --- | --- | --- | --- |
| 7d_1 | 2.21% | 0.73% | 4.44% | 5.19% | 86.85% | 0.59% |
| 7d_2 | 2.98% | 1.06% | 4.71% | 5.28% | 85.49% | 0.48% |
| 7d_3 | 1.99% | 1.02% | 5.66% | 4.97% | 85.80% | 0.56% |
| 7d_4 | 4.59% | 5.62% | 4.30% | 4.98% | 79.95% | 0.57% |
| 7d_5 | 2.38% | 0.86% | 3.59% | 5.23% | 87.37% | 0.56% |
| 4d_1 | 4.90% | 1.91% | 6.00% | 5.25% | 81.26% | 0.68% |
| 4d_2 | 4.22% | 1.55% | 4.57% | 5.55% | 83.49% | 0.62% |
| 4d_3 | 5.08% | 2.02% | 6.13% | 5.40% | 80.72% | 0.65% |
| 4d_4 | 5.53% | 2.37% | 5.15% | 5.30% | 80.97% | 0.67% |
| 4d_5 | 4.78% | 1.78% | 4.55% | 5.19% | 83.01% | 0.68% |
| 3d_1 | 5.16% | 1.94% | 4.94% | 5.42% | 81.94% | 0.60% |
| 3d_2 | 4.37% | 1.78% | 4.40% | 5.67% | 83.15% | 0.63% |
| 3d_3 | 5.56% | 2.24% | 5.13% | 5.29% | 81.09% | 0.69% |
| 3d_4 | 4.30% | 1.69% | 3.98% | 5.56% | 83.85% | 0.60% |
| 3d_5 | 5.77% | 2.11% | 5.13% | 4.94% | 81.42% | 0.62% |
| 2d_1 | 8.13% | 3.13% | 6.12% | 5.27% | 76.69% | 0.66% |
| 2d_2 | 4.53% | 1.81% | 4.08% | 5.65% | 83.12% | 0.81% |
| 2d_3 | 7.61% | 3.36% | 7.14% | 5.13% | 76.16% | 0.60% |
| 2d_4 | 5.12% | 1.91% | 4.83% | 5.24% | 82.12% | 0.77% |
| 2d_5 | 5.67% | 2.17% | 4.25% | 5.54% | 81.74% | 0.63% |
| 24h_1 | 6.20% | 2.30% | 4.73% | 5.11% | 81.02% | 0.64% |
| 24h_2 | 4.96% | 1.97% | 4.54% | 5.54% | 82.38% | 0.62% |
| 24h_3 | 6.61% | 2.57% | 5.38% | 5.29% | 79.49% | 0.65% |
| 24h_4 | 4.90% | 1.91% | 3.55% | 5.44% | 83.48% | 0.72% |
| 24h_5 | 5.30% | 1.99% | 3.74% | 5.53% | 82.72% | 0.72% |
| 7h_1 | 6.26% | 2.43% | 6.37% | 5.16% | 79.12% | 0.66% |
| 7h_2 | 5.54% | 4.73% | 4.04% | 10.10% | 74.99% | 0.59% |
| 7h_3 | 6.13% | 2.27% | 5.33% | 5.01% | 80.54% | 0.71% |
| 7h_4 | 5.46% | 1.84% | 3.87% | 5.45% | 82.71% | 0.68% |
| 7h_5 | 5.67% | 2.12% | 4.44% | 5.14% | 81.91% | 0.71% |
| 1h_1 | 8.10% | 2.87% | 5.79% | 4.75% | 77.87% | 0.62% |
| 1h_2 | 6.38% | 2.42% | 4.82% | 5.13% | 80.51% | 0.73% |
| 1h_3 | 6.30% | 2.40% | 5.11% | 5.49% | 79.96% | 0.74% |
| 1h_4 | 6.01% | 2.30% | 4.36% | 5.31% | 81.27% | 0.74% |
| 1h_5 | 6.76% | 2.51% | 5.20% | 5.42% | 79.36% | 0.75% |
| 5min_1 | 9.40% | 3.52% | 8.52% | 5.82% | 72.17% | 0.57% |
| 5min_2 | 6.69% | 2.63% | 4.97% | 5.27% | 79.72% | 0.72% |
| 5min_3 | 6.36% | 2.47% | 5.56% | 5.75% | 79.20% | 0.67% |
| 5min_4 | 5.74% | 2.26% | 4.04% | 5.42% | 81.79% | 0.74% |
| 5min_5 | 6.45% | 2.44% | 4.48% | 5.64% | 80.23% | 0.77% |

**Table S2F:** Glycosylation profiles for the PK study after **subcutaneous injection** of **ST3 mAb1**Relative intensities for the quantified glycoforms are given in percentage separately for each time point and each animal (five biological replicates).

| Time point_animal | G0F | G1F | G2FS1 | G2S2 | G2FS2 | H6N4F1S2 |
| --- | --- | --- | --- | --- | --- | --- |
| 10d_1 | 2.39% | 0.19% | 6.83% | 4.24% | 85.29% | 1.06% |
| 10d_2 |  |  |  |  |  |  |
| 10d_3 | 2.33% | 1.59% | 5.87% | 4.39% | 85.00% | 0.82% |
| 10d_4 | 1.18% | 0.16% | 6.38% | 3.92% | 86.01% | 2.35% |
| 10d_5 | 1.92% | 0.82% | 4.84% | 4.90% | 86.53% | 0.99% |
| 7d_1 | 2.57% | 1.16% | 5.20% | 5.31% | 84.97% | 0.79% |
| 7d_2 | 2.53% | 1.25% | 5.51% | 5.55% | 84.84% | 0.32% |
| 7d_3 | 2.33% | 1.70% | 3.88% | 5.00% | 86.18% | 0.90% |
| 7d_4 | 3.08% | 1.77% | 5.41% | 4.94% | 84.18% | 0.61% |
| 7d_5 | 3.67% | 2.02% | 6.12% | 4.95% | 82.53% | 0.72% |
| 4d_1 | 3.91% | 2.08% | 5.81% | 4.83% | 82.69% | 0.68% |
| 4d_2 | 3.77% | 2.05% | 4.91% | 5.11% | 83.44% | 0.72% |
| 4d_3 | 4.07% | 2.07% | 4.81% | 5.12% | 83.23% | 0.69% |
| 4d_4 | 4.56% | 2.29% | 5.87% | 4.83% | 81.74% | 0.71% |
| 4d_5 | 3.13% | 0.83% | 6.69% | 4.40% | 84.82% | 0.13% |
| 3d_1 | 4.12% | 2.03% | 5.22% | 5.06% | 82.82% | 0.75% |
| 3d_2 | 3.59% | 2.00% | 2.83% | 5.12% | 85.72% | 0.73% |
| 3d_3 | 4.36% | 2.42% | 5.26% | 4.85% | 82.38% | 0.72% |
| 3d_4 | 4.49% | 2.44% | 5.77% | 4.77% | 81.81% | 0.73% |
| 3d_5 | 3.16% | 1.67% | 3.28% | 5.41% | 85.76% | 0.72% |
| 2d_1 | 4.29% | 2.27% | 4.18% | 5.17% | 83.32% | 0.76% |
| 2d_2 | 4.30% | 2.20% | 4.69% | 5.27% | 82.79% | 0.74% |
| 2d_3 | 4.90% | 2.62% | 5.18% | 5.11% | 81.45% | 0.74% |
| 2d_4 | 4.63% | 2.39% | 5.25% | 4.85% | 82.11% | 0.77% |
| 2d_5 | 4.71% | 2.42% | 5.63% | 5.48% | 81.42% | 0.34% |
| 24h_1 | 4.52% | 2.46% | 4.51% | 5.57% | 82.14% | 0.80% |
| 24h_2 | 3.94% | 2.10% | 3.71% | 5.31% | 84.19% | 0.74% |
| 24h_3 | 4.25% | 2.34% | 3.25% | 5.67% | 83.73% | 0.76% |
| 24h_4 | 4.11% | 2.27% | 3.50% | 5.37% | 83.98% | 0.76% |
| 24h_5 | 5.95% | 3.17% | 5.23% | 4.66% | 80.25% | 0.74% |
| 7h_1 | 6.57% | 3.40% | 6.57% | 4.41% | 78.38% | 0.67% |
| 7h_2 | 6.41% | 3.29% | 7.27% | 4.80% | 77.57% | 0.66% |
| 7h_3 | 5.70% | 3.06% | 5.57% | 4.75% | 80.17% | 0.75% |
| 7h_4 | 4.30% | 2.50% | 4.48% | 5.37% | 82.79% | 0.57% |
| 7h_5 | 4.86% | 2.64% | 4.00% | 5.20% | 82.53% | 0.77% |
| 2h_1 | 5.51% | 2.87% | 5.79% | 4.65% | 80.51% | 0.66% |
| 2h_2 | 4.60% | 2.11% | 5.57% | 4.93% | 82.10% | 0.68% |
| 2h_3 | 5.77% | 2.79% | 5.62% | 4.86% | 80.29% | 0.67% |
| 2h_4 | 5.65% | 2.89% | 5.48% | 4.90% | 80.32% | 0.77% |
| 2h_5 | 4.61% | 1.81% | 5.49% | 4.73% | 82.74% | 0.62% |

**Table S2G:** Glycosylation profiles for the PK study after **subcutaneous injection** of **CHO mAb2**Relative intensities for the quantified glycoforms are given in percentage separately for each time point and each animal (five biological replicates).

| Animal_time point | Man5 | G0F-N | G0 | G0F | G1 | G1F | G2F |
| --- | --- | --- | --- | --- | --- | --- | --- |
| 101F1_2h | 0.96% | 1.91% | 1.31% | 42.53% | 1.04% | 44.01% | 8.25% |
| 102F1_2h | 0.96% | 1.87% | 1.28% | 42.51% | 0.95% | 44.15% | 8.28% |
| 103F1_2h | 0.96% | 1.91% | 1.32% | 42.69% | 0.89% | 44.06% | 8.18% |
| 104F1_2h | 0.95% | 1.90% | 1.26% | 42.68% | 0.92% | 44.13% | 8.16% |
| 105F1_2h | 0.95% | 1.99% | 1.25% | 42.55% | 0.85% | 44.25% | 8.16% |
| 101F1_7h | 1.02% | 2.01% | 1.33% | 42.44% | 1.12% | 43.71% | 8.37% |
| 102F1_7h | 1.02% | 2.10% | 1.35% | 42.42% | 0.86% | 43.88% | 8.38% |
| 103F1_7h | 1.03% | 1.94% | 1.30% | 42.53% | 0.80% | 44.02% | 8.38% |
| 104F1_7h | 1.03% | 2.04% | 1.36% | 42.50% | 0.80% | 43.88% | 8.39% |
| 105F1_7h | 1.02% | 2.00% | 1.41% | 42.45% | 0.81% | 43.84% | 8.47% |
| 101F1_24h | 0.98% | 2.00% | 1.35% | 42.74% | 1.07% | 43.52% | 8.33% |
| 102F1_24h | 0.99% | 2.02% | 1.29% | 42.57% | 0.80% | 43.91% | 8.41% |
| 103F1_24h | 1.02% | 1.99% | 1.40% | 42.57% | 0.81% | 43.75% | 8.45% |
| 104F1_24h | 0.87% | 2.01% | 1.33% | 42.66% | 1.03% | 43.73% | 8.38% |
| 105F1_24h | 0.96% | 1.99% | 1.35% | 42.41% | 0.96% | 43.88% | 8.45% |
| 101F1_2d | 0.97% | 1.98% | 1.34% | 42.47% | 0.91% | 43.85% | 8.47% |
| 102F1_2d | 0.97% | 1.97% | 1.31% | 42.65% | 0.92% | 43.88% | 8.30% |
| 103F1_2d | 1.02% | 1.99% | 1.39% | 42.51% | 1.08% | 43.55% | 8.45% |
| 104F1_2d | 0.80% | 2.08% | 1.39% | 42.64% | 0.89% | 43.72% | 8.48% |
| 105F1_2d | 0.98% | 2.03% | 1.37% | 42.36% | 1.06% | 43.65% | 8.54% |
| 101F1_3d | 0.94% | 2.02% | 1.40% | 42.46% | 1.05% | 43.50% | 8.63% |
| 102F1_3d | 1.00% | 2.04% | 1.39% | 42.50% | 0.80% | 43.76% | 8.52% |
| 103F1_3d | 0.99% | 2.04% | 1.44% | 42.52% | 1.05% | 43.40% | 8.57% |
| 104F1_3d | 0.75% | 2.00% | 1.40% | 42.37% | 0.94% | 43.91% | 8.62% |
| 105F1_3d | 0.93% | 2.04% | 1.41% | 42.54% | 1.17% | 43.44% | 8.47% |
| 101F1_4d | 0.92% | 2.04% | 1.40% | 42.51% | 1.08% | 43.60% | 8.47% |
| 102F1_4d | 0.96% | 2.04% | 1.36% | 42.52% | 1.10% | 43.51% | 8.51% |
| 103F1_4d | 0.96% | 2.06% | 1.38% | 42.38% | 1.03% | 43.74% | 8.46% |
| 104F1_4d | 0.72% | 2.06% | 1.34% | 42.64% | 1.12% | 43.74% | 8.37% |
| 105F1_4d | 0.90% | 2.02% | 1.35% | 42.66% | 1.13% | 43.60% | 8.34% |
| 101F1_7d | 0.84% | 2.00% | 1.34% | 42.43% | 0.90% | 44.12% | 8.38% |
| 102F1_7d | 0.90% | 1.97% | 1.33% | 42.54% | 1.13% | 43.65% | 8.47% |
| 103F1_7d | 0.90% | 2.02% | 1.37% | 42.47% | 0.97% | 43.73% | 8.54% |
| 104F1_7d | 0.66% | 1.98% | 1.35% | 42.57% | 0.88% | 44.06% | 8.50% |
| 105F1_7d | 0.83% | 1.98% | 1.39% | 42.62% | 0.92% | 43.86% | 8.41% |
| 101F1_10d | 0.80% | 1.97% | 1.31% | 42.51% | 1.07% | 43.85% | 8.48% |
| 102F1_10d | 0.82% | 1.97% | 1.34% | 42.55% | 1.13% | 43.82% | 8.37% |
| 103F1_10d | 0.78% | 1.94% | 1.30% | 42.58% | 0.98% | 44.02% | 8.41% |
| 104F1_10d | 0.58% | 1.94% | 1.31% | 42.54% | 1.01% | 44.21% | 8.42% |
| 105F1_10d | 0.76% | 2.02% | 1.35% | 42.61% | 1.04% | 43.77% | 8.44% |
| 101F1_14d | 0.54% | 1.90% | 1.28% | 42.88% | 0.82% | 44.30% | 8.29% |
| 102F1_14d | 0.66% | 1.93% | 1.32% | 42.69% | 0.83% | 44.23% | 8.34% |
| 103F1_14d | 0.71% | 1.91% | 1.33% | 42.55% | 0.91% | 44.25% | 8.34% |
| 104F1_14d | 0.53% | 1.96% | 1.26% | 42.59% | 0.86% | 44.23% | 8.57% |
| 105F1_14d | 0.66% | 1.95% | 1.35% | 42.57% | 0.86% | 44.23% | 8.38% |
| 101F1_21d | 0.49% | 1.84% | 1.26% | 42.69% | 0.93% | 44.35% | 8.45% |
| 102F1_21d | 0.49% | 1.86% | 1.12% | 42.54% | 0.84% | 44.57% | 8.59% |
| 103F1_21d | 0.53% | 1.89% | 1.26% | 42.88% | 0.87% | 44.21% | 8.35% |
| 104F1_21d | 0.57% | 1.92% | 1.42% | 42.42% | 0.98% | 44.22% | 8.47% |
| 105F1_21d | 0.47% | 1.79% | 1.25% | 43.31% | 0.90% | 44.07% | 8.20% |
| 101F1_28d | 0.33% | 2.11% | 1.25% | 42.90% | 0.92% | 43.98% | 8.52% |
| 102F1_28d |  |  |  |  |  |  |  |
| 103F1_28d | 0.43% | 1.85% | 1.22% | 42.92% | 0.94% | 44.54% | 8.10% |
| 104F1_28d | 0.33% | 1.82% | 1.24% | 42.92% | 0.96% | 44.63% | 8.10% |
| 105F1_28d | 0.37% | 1.85% | 1.33% | 43.51% | 0.93% | 43.86% | 8.14% |
| 101F1_35d | 0.40% | 1.96% | 1.23% | 42.62% | 1.00% | 44.27% | 8.54% |
| 102F1_35d |  |  |  |  |  |  |  |
| 103F1_35d | 0.38% | 1.79% | 1.27% | 42.49% | 0.90% | 44.66% | 8.53% |
| 104F1_35d | 0.36% | 1.78% | 1.29% | 42.80% | 0.84% | 44.53% | 8.40% |
| 105F1_35d | 0.45% | 1.88% | 1.39% | 43.77% | 0.85% | 43.47% | 8.18% |
| 101F1_42d | 0.29% | 1.95% | 1.29% | 42.35% | 1.31% | 44.31% | 8.50% |
| 102F1_42d |  |  |  |  |  |  |  |
| 103F1_42d | 0.32% | 1.83% | 1.19% | 42.25% | 0.86% | 45.01% | 8.54% |
| 104F1_42d | 0.32% | 1.87% | 1.36% | 42.49% | 0.90% | 44.62% | 8.43% |
| 105F1_42d | 0.35% | 1.95% | 1.32% | 44.40% | 0.79% | 43.09% | 8.10% |

**Table S2H:** Glycosylation profiles for the PK study after **subcutaneous injection** of **ST3 mAb2**Relative intensities for the quantified glycoforms are given in percentage separately for each time point and each animal (five biological replicates).

| Animal_time point | Man5 | G1S-N | G1FS-N | G2F | G2S1 | G2FS1 | G2S2 | G2FS2 |
| --- | --- | --- | --- | --- | --- | --- | --- | --- |
| 201F1_2h | 0.65% | 0.27% | 1.87% | 2.60% | 0.61% | 27.22% | 1.30% | 65.47% |
| 202F1_2h | 0.63% | 0.26% | 1.93% | 2.84% | 0.62% | 28.31% | 1.29% | 64.12% |
| 203F1_2h | 0.66% | 0.22% | 1.96% | 2.26% | 0.58% | 26.57% | 1.33% | 66.41% |
| 204F1_2h | 0.71% | 0.28% | 1.99% | 2.40% | 0.57% | 26.79% | 1.33% | 65.93% |
| 205F1_2h | 0.64% | 0.29% | 1.86% | 2.43% | 0.57% | 26.72% | 1.34% | 66.17% |
| 201F1_7h | 0.74% | 0.24% | 2.02% | 2.80% | 0.61% | 28.07% | 1.41% | 64.12% |
| 202F1_7h | 0.73% | 0.27% | 2.17% | 3.16% | 0.64% | 29.37% | 1.44% | 62.22% |
| 203F1_7h | 0.70% | 0.25% | 1.97% | 2.75% | 0.59% | 28.35% | 1.35% | 64.03% |
| 204F1_7h | 0.74% | 0.26% | 1.99% | 2.87% | 0.62% | 28.71% | 1.38% | 63.43% |
| 205F1_7h | 0.73% | 0.28% | 2.03% | 2.69% | 0.63% | 28.04% | 1.33% | 64.27% |
| 201F1_24h | 0.67% | 0.24% | 2.01% | 3.15% | 0.62% | 29.54% | 1.34% | 62.42% |
| 202F1_24h | 0.60% | 0.26% | 1.98% | 2.87% | 0.58% | 28.39% | 1.33% | 64.00% |
| 203F1_24h | 0.72% | 0.27% | 2.04% | 2.88% | 0.60% | 28.49% | 1.39% | 63.61% |
| 204F1_24h | 0.76% | 0.26% | 2.01% | 3.04% | 0.62% | 29.16% | 1.34% | 62.81% |
| 205F1_24h | 0.69% | 0.25% | 1.94% | 2.93% | 0.61% | 28.67% | 1.35% | 63.55% |
| 201F1_2d | 0.68% | 0.27% | 2.06% | 2.84% | 0.61% | 28.68% | 1.35% | 63.51% |
| 202F1_2d | 0.59% | 0.25% | 1.96% | 3.08% | 0.62% | 29.36% | 1.35% | 62.78% |
| 203F1_2d | 0.69% | 0.27% | 2.02% | 2.79% | 0.61% | 28.59% | 1.40% | 63.65% |
| 204F1_2d | 0.78% | 0.28% | 2.01% | 2.91% | 0.59% | 28.67% | 1.41% | 63.37% |
| 205F1_2d | 0.66% | 0.25% | 1.94% | 2.96% | 0.65% | 29.20% | 1.39% | 62.96% |
| 201F1_3d | 0.57% | 0.24% | 2.05% | 2.81% | 0.61% | 28.64% | 1.38% | 63.70% |
| 202F1_3d | 0.54% | 0.24% | 1.98% | 2.79% | 0.63% | 28.23% | 1.37% | 64.22% |
| 203F1_3d | 0.65% | 0.25% | 1.93% | 3.00% | 0.66% | 29.03% | 1.36% | 63.12% |
| 204F1_3d | 0.69% | 0.25% | 2.00% | 2.92% | 0.62% | 28.54% | 1.42% | 63.56% |
| 205F1_3d | 0.66% | 0.25% | 2.04% | 2.97% | 0.64% | 29.07% | 1.41% | 62.95% |
| 201F1_4d | 0.51% | 0.22% | 1.99% | 3.03% | 0.64% | 29.27% | 1.41% | 62.92% |
| 202F1_4d | 0.48% | 0.24% | 2.02% | 2.97% | 0.64% | 29.09% | 1.39% | 63.17% |
| 203F1_4d | 0.69% | 0.25% | 1.99% | 2.79% | 0.65% | 28.29% | 1.41% | 63.95% |
| 204F1_4d | 0.70% | 0.30% | 2.01% | 3.06% | 0.65% | 28.82% | 1.59% | 62.86% |
| 205F1_4d | 0.65% | 0.25% | 1.94% | 3.08% | 0.66% | 29.33% | 1.38% | 62.71% |
| 201F1_7d | 0.50% | 0.27% | 2.11% | 3.00% | 0.62% | 29.05% | 1.54% | 62.90% |
| 202F1_7d | 0.51% | 0.24% | 2.07% | 3.21% | 0.64% | 29.99% | 1.34% | 62.00% |
| 203F1_7d | 0.60% | 0.25% | 1.95% | 3.11% | 0.66% | 29.48% | 1.39% | 62.57% |
| 204F1_7d | 0.66% | 0.25% | 2.02% | 3.19% | 0.68% | 29.52% | 1.37% | 62.31% |
| 205F1_7d | 0.55% | 0.27% | 1.96% | 2.79% | 0.61% | 28.25% | 1.37% | 64.20% |
| 201F1_20d | 0.44% | 0.24% | 2.01% | 2.84% | 0.62% | 28.75% | 1.38% | 63.70% |
| 202F1_20d | 0.43% | 0.24% | 2.01% | 2.93% | 0.66% | 28.96% | 1.38% | 63.38% |
| 203F1_20d | 0.55% | 0.24% | 2.04% | 2.99% | 0.61% | 29.18% | 1.35% | 63.03% |
| 204F1_20d | 0.59% | 0.26% | 2.03% | 3.10% | 0.66% | 29.13% | 1.40% | 62.82% |
| 205F1_20d | 0.50% | 0.24% | 1.99% | 2.79% | 0.60% | 28.54% | 1.34% | 64.00% |
| 201F1_14d | 0.35% | 0.23% | 1.91% | 2.65% | 0.63% | 28.17% | 1.36% | 64.69% |
| 202F1_14d | 0.39% | 0.23% | 1.94% | 3.01% | 0.58% | 29.15% | 1.38% | 63.32% |
| 203F1_14d | 0.51% | 0.24% | 2.01% | 2.69% | 0.61% | 27.97% | 1.37% | 64.59% |
| 204F1_14d | 0.54% | 0.23% | 1.95% | 3.11% | 0.67% | 29.33% | 1.41% | 62.75% |
| 205F1_14d | 0.45% | 0.22% | 1.95% | 2.87% | 0.65% | 28.77% | 1.38% | 63.72% |
| 201F1_21d | 0.37% | 0.21% | 1.92% | 2.73% | 0.71% | 27.68% | 1.43% | 64.94% |
| 202F1_21d | 0.37% | 0.19% | 1.94% | 2.65% | 0.66% | 27.42% | 1.38% | 65.39% |
| 203F1_21d | 0.41% | 0.23% | 1.88% | 2.69% | 0.58% | 28.16% | 1.35% | 64.70% |
| 204F1_21d | 0.47% | 0.23% | 1.91% | 2.86% | 0.71% | 28.53% | 1.42% | 63.86% |
| 205F1_21d | 0.40% | 0.24% | 1.85% | 2.57% | 0.82% | 26.62% | 1.41% | 66.10% |
| 201F1_28d | 0.41% | 0.27% | 2.41% | 3.49% | 0.82% | 28.92% | 1.52% | 62.16% |
| 202F1_28d | 0.36% | 0.21% | 1.95% | 2.66% | 0.70% | 27.57% | 1.37% | 65.18% |
| 203F1_28d | 0.34% | 0.22% | 1.84% | 2.60% | 0.56% | 27.05% | 1.33% | 66.06% |
| 204F1_28d | 0.39% | 0.25% | 1.90% | 2.73% | 0.58% | 27.65% | 1.35% | 65.15% |
| 205F1_28d |  |  |  |  |  |  |  |  |
| 201F1_35d | 0.32% | 0.20% | 1.95% | 2.97% | 0.77% | 29.00% | 1.46% | 63.33% |
| 202F1_35d | 0.31% | 0.24% | 1.89% | 2.64% | 0.70% | 27.84% | 1.46% | 64.93% |
| 203F1_35d | 0.35% | 0.21% | 1.90% | 2.68% | 0.71% | 27.82% | 1.41% | 64.93% |
| 204F1_35d | 0.42% | 0.25% | 1.94% | 2.73% | 0.68% | 27.67% | 1.39% | 64.90% |
| 205F1_35d |  |  |  |  |  |  |  |  |
| 201F1_42d | 0.33% | 0.19% | 1.85% | 2.70% | 0.70% | 27.26% | 1.47% | 65.50% |
| 202F1_42d | 0.39% | 0.21% | 1.90% | 2.63% | 0.78% | 27.23% | 1.42% | 65.43% |
| 203F1_42d | 0.39% | 0.24% | 1.95% | 2.64% | 0.73% | 27.42% | 1.45% | 65.18% |
| 204F1_42d | 0.40% | 0.23% | 1.92% | 2.53% | 0.78% | 26.89% | 1.42% | 65.83% |
| 205F1_42d |  |  |  |  |  |  |  |  |

**Table S3A:** Individual glycoform concentrations for the PK study after **intravenous injection** of **CHO mAb1**Concentrations of individual glycoforms are calculated from the total antibody concentration determined by ELISA (total) and the glycosylation profiles (Table S10A). They are given in ng/mL separately for each time point and each animal (five biological replicates).

| Time point_ animal | Total | Man5 | G0F-N | G0 | G0F | G1 | G1F | G2F |
| --- | --- | --- | --- | --- | --- | --- | --- | --- |
| 4d_1 | 1500 | 31 | 80 | 60 | 908 | 24 | 364 | 33 |
| 4d_2 | 2060 | 34 | 98 | 79 | 1264 | 33 | 506 | 46 |
| 4d_3 | 1850 | 40 | 100 | 72 | 1117 | 25 | 452 | 43 |
| 4d_4 | 2040 | 30 | 99 | 79 | 1256 | 31 | 498 | 46 |
| 4d_5 | 1730 | 28 | 80 | 66 | 1059 | 27 | 431 | 40 |
| 3d_1 | 2080 | 36 | 98 | 76 | 1286 | 30 | 506 | 48 |
| 3d_2 | 2810 | 59 | 143 | 110 | 1707 | 44 | 683 | 66 |
| 3d_3 | 2990 | 61 | 145 | 115 | 1834 | 40 | 730 | 65 |
| 3d_4 | 3220 | 60 | 160 | 118 | 1978 | 45 | 787 | 73 |
| 3d_5 | 2460 | 49 | 127 | 95 | 1502 | 35 | 597 | 55 |
| 2d_1 | 3670 | 68 | 179 | 133 | 2261 | 52 | 894 | 83 |
| 2d_2 | 4010 | 85 | 200 | 152 | 2453 | 59 | 968 | 91 |
| 2d_3 | 4770 | 91 | 225 | 172 | 2951 | 67 | 1156 | 108 |
| 2d_4 | 4120 | 85 | 230 | 164 | 2515 | 56 | 982 | 89 |
| 2d_5 | 3450 | 67 | 179 | 134 | 2126 | 47 | 824 | 73 |
| 24h_1 | 6430 | 151 | 371 | 250 | 3927 | 82 | 1512 | 137 |
| 24h_2 | 5510 | 108 | 274 | 206 | 3411 | 76 | 1313 | 122 |
| 24h_3 | 6620 | 128 | 329 | 239 | 4088 | 91 | 1594 | 150 |
| 24h_4 | 6730 | 137 | 320 | 256 | 4146 | 92 | 1624 | 155 |
| 24h_5 | 5190 | 105 | 279 | 203 | 3187 | 74 | 1231 | 112 |
| 7h_1 | 9400 | 209 | 500 | 361 | 5779 | 125 | 2219 | 206 |
| 7h_2 | 9740 | 226 | 533 | 381 | 5956 | 151 | 2280 | 215 |
| 7h_3 | 10200 | 238 | 510 | 392 | 6219 | 135 | 2470 | 236 |
| 7h_4 | 11000 | 203 | 529 | 416 | 6831 | 164 | 2614 | 242 |
| 7h_5 | 8020 | 189 | 432 | 309 | 4893 | 111 | 1907 | 179 |
| 1h_1 | 12000 | 265 | 626 | 455 | 7409 | 160 | 2824 | 260 |
| 1h_2 | 12800 | 258 | 651 | 478 | 8010 | 164 | 2971 | 268 |
| 1h_3 | 12100 | 277 | 673 | 444 | 7476 | 160 | 2814 | 256 |
| 1h_4 | 13200 | 244 | 690 | 457 | 8206 | 178 | 3139 | 286 |
| 1h_5 | 9660 | 175 | 479 | 339 | 6071 | 125 | 2267 | 203 |
| 5min_1 | 12600 | 326 | 721 | 488 | 7689 | 161 | 2941 | 273 |
| 5min_2 | 13400 | 367 | 666 | 522 | 8209 | 173 | 3160 | 303 |
| 5min_3 | 14800 | 399 | 698 | 566 | 9075 | 199 | 3517 | 346 |
| 5min_4 | 25300 | 687 | 1425 | 946 | 15369 | 331 | 5980 | 561 |
| 5min_5 | 12700 | 351 | 716 | 464 | 7684 | 166 | 3027 | 291 |

**Table S3B:** Individual glycoform concentrations for the PK study after **subcutaneous injection** of **CHO mAb1**Concentrations of individual glycoforms are calculated from the total antibody concentration determined by ELISA (total) and the glycosylation profiles (Table S10B). They are given in ng/mL separately for each time point and each animal (five biological replicates).

| Time point_ animal | Total | Man5 | G0F-N | G0 | G0F | G1 | G1F | G2F |
| --- | --- | --- | --- | --- | --- | --- | --- | --- |
| 7d_1 | 572 | 9.5 | 36 | 39 | 318 | 12 | 143 | 15 |
| 7d_2 | 484 | 4.1 | 29 | 29 | 277 | 7 | 127 | 12 |
| 7d_3 | 677 | 9.1 | 34 | 39 | 395 | 12 | 173 | 16 |
| 7d_4 | 291 | 3.5 | 18 | 20 | 160 | 5 | 76 | 8 |
| 7d_5 | 499 | 7.7 | 23 | 31 | 266 | 9 | 145 | 17 |
| 4d_1 | 1570 | 9.4 | 57 | 74 | 895 | 25 | 463 | 47 |
| 4d_2 | 1730 | 10.5 | 55 | 79 | 982 | 28 | 521 | 54 |
| 4d_3 | 1280 | 7.9 | 52 | 63 | 729 | 20 | 372 | 38 |
| 4d_4 | 1150 | 3.8 | 41 | 60 | 657 | 19 | 339 | 29 |
| 4d_5 | 1950 | 30.9 | 106 | 105 | 1058 | 29 | 563 | 58 |
| 3d_1 | 2100 | 6.1 | 85 | 112 | 1268 | 30 | 559 | 39 |
| 3d_2 | 2400 | 18.1 | 91 | 119 | 1459 | 40 | 623 | 49 |
| 3d_3 | 2010 | 16.0 | 94 | 98 | 1143 | 26 | 579 | 55 |
| 3d_4 | 1660 | 11.7 | 72 | 85 | 939 | 26 | 481 | 45 |
| 3d_5 | 3260 | 33.1 | 122 | 151 | 1828 | 51 | 977 | 99 |
| 2d_1 | 2570 | 20.7 | 85 | 121 | 1441 | 39 | 780 | 83 |
| 2d_2 | 3000 | 20.8 | 93 | 125 | 1710 | 50 | 903 | 97 |
| 2d_3 | 2400 | 17.4 | 69 | 99 | 1378 | 38 | 723 | 77 |
| 2d_4 | 2400 | 13.7 | 85 | 115 | 1365 | 36 | 713 | 72 |
| 2d_5 | 4450 | 38.6 | 191 | 211 | 2533 | 66 | 1283 | 127 |
| 24h_1 | 2730 | 23.7 | 110 | 129 | 1579 | 43 | 774 | 71 |
| 24h_2 | 3390 | 26.5 | 136 | 153 | 1958 | 48 | 979 | 90 |
| 24h_3 | 2560 | 28.3 | 80 | 117 | 1439 | 41 | 773 | 81 |
| 24h_4 | 3540 | 31.0 | 149 | 168 | 2018 | 59 | 1014 | 100 |
| 24h_5 | 5820 | 60.9 | 171 | 253 | 3263 | 95 | 1780 | 196 |
| 7h_1 | 2190 | 21.6 | 76 | 95 | 1249 | 37 | 646 | 65 |
| 7h_2 | 2000 | 14.8 | 61 | 85 | 1151 | 33 | 594 | 61 |
| 7h_3 | 2540 | 11.6 | 78 | 110 | 1473 | 40 | 754 | 74 |
| 7h_4 | 3410 | 30.5 | 132 | 154 | 1989 | 56 | 958 | 90 |
| 7h_5 | 5680 | 34.0 | 227 | 257 | 3298 | 77 | 1644 | 144 |
| 2h_1 | 732 | 8.4 | 28 | 40 | 417 | 12 | 208 | 17 |
| 2h_2 | 447 | 0.0 | 26 | 28 | 256 | 1.3 | 126 | 10 |
| 2h_3 | 699 |  |  |  |  |  |  |  |
| 2h_4 | 1500 | 6.2 | 60 | 73 | 869 | 19 | 435 | 37 |
| 2h_5 | 3560 | 45.9 | 137 | 174 | 2021 | 61 | 1023 | 99 |

**Table S3C:** Individual glycoform concentrations for the PK study after **intravenous injection** of **M5 mAb1**Concentrations of individual glycoforms are calculated from the total antibody concentration determined by ELISA (total) and the glycosylation profiles (Table S10C). They are given in ng/mL separately for each time point and each animal (five biological replicates).

| Time point_animal | Total | Man5 | G0F | G1F |
| --- | --- | --- | --- | --- |
| 4d_1 | 832 | 741 | 47 | 44 |
| 4d_2 | 1510 | 1340 | 86 | 84 |
| 4d_3 | 1360 | 1218 | 74 | 68 |
| 4d_4 | 839 | 766 | 39 | 34 |
| 4d_5 | 1580 | 1432 | 76 | 72 |
| 3d_1 | 1220 | 1103 | 59 | 58 |
| 3d_2 | 2590 | 2315 | 139 | 136 |
| 3d_3 | 1790 | 1616 | 91 | 83 |
| 3d_4 | 1430 | 1304 | 63 | 63 |
| 3d_5 | 2290 | 2079 | 106 | 105 |
| 2d_1 | 1940 | 1760 | 90 | 90 |
| 2d_2 | 3630 | 3291 | 169 | 170 |
| 2d_3 | 3260 | 2966 | 148 | 146 |
| 2d_4 | 2890 | 2645 | 121 | 124 |
| 2d_5 | 3540 | 3260 | 144 | 137 |
| 24h_1 | 3240 | 2965 | 131 | 144 |
| 24h_2 | 5280 | 4851 | 224 | 205 |
| 24h_3 | 5520 | 5036 | 230 | 254 |
| 24h_4 | 3540 | 3288 | 131 | 121 |
| 24h_5 | 5610 | 5153 | 220 | 237 |
| 7h_1 | 5010 | 4652 | 179 | 179 |
| 7h_2 | 6980 | 6436 | 244 | 300 |
| 7h_3 | 8980 | 8348 | 316 | 316 |
| 7h_4 | 5200 | 4806 | 189 | 205 |
| 7h_5 | 6050 | 5599 | 222 | 229 |
| 1h_1 | 6360 | 5896 | 238 | 225 |
| 1h_2 | 9350 | 8702 | 330 | 318 |
| 1h_3 | 11700 | 10826 | 455 | 419 |
| 1h_4 | 8110 | 7539 | 296 | 275 |
| 1h_5 | 12900 | 11971 | 485 | 444 |
| 5min_1 | 6450 | 5989 | 240 | 221 |
| 5min_2 | 15300 | 14138 | 604 | 559 |
| 5min_3 | 13800 | 12661 | 587 | 552 |
| 5min_4 | 11600 | 10776 | 419 | 405 |
| 5min_5 | 12900 | 11903 | 516 | 481 |

**Table S3D:** Individual glycoform concentrations for the PK study after **subcutaneous injection** of **M5 mAb1**Concentrations of individual glycoforms are calculated from the total antibody concentration determined by ELISA (total) and the glycosylation profiles (Table S10D). They are given in ng/mL separately for each time point and each animal (five biological replicates).

| Time point_animal | Total | Man5 | G0F | G1F |
| --- | --- | --- | --- | --- |
| 10d_1 | 87.5 | 73 | 7.1 | 6.9 |
| 10d_2 | 89.7 | 66 | 14.0 | 10.1 |
| 10d_3 | 122 | 102 | 9.8 | 9.8 |
| 10d_4 | 183 | 156 | 13.7 | 12.8 |
| 10d_5 | 53.1 | 43 | 5.5 | 4.7 |
| 7d_1 | 522 | 462 | 32 | 27 |
| 7d_2 | 287 | 236 | 28 | 23 |
| 7d_3 | 470 | 417 | 28 | 25 |
| 7d_4 | 708 | 637 | 35 | 36 |
| 7d_5 | 268 | 216 | 26 | 26 |
| 4d_1 | 1610 | 1372 | 108 | 130 |
| 4d_2 | 1420 | 1235 | 88 | 97 |
| 4d_3 | 1510 | 1340 | 82 | 88 |
| 4d_4 | 2190 | 1967 | 102 | 121 |
| 4d_5 | 1190 | 1045 | 73 | 72 |
| 3d_1 | 2520 | 2292 | 114 | 114 |
| 3d_2 | 1690 | 1495 | 94 | 101 |
| 3d_3 | 2120 | 1919 | 94 | 107 |
| 3d_4 | 2910 | 2648 | 117 | 146 |
| 3d_5 | 1980 | 1776 | 99 | 106 |
| 2d_1 | 3580 | 3243 | 158 | 179 |
| 2d_2 | 2270 | 2056 | 113 | 100 |
| 2d_3 | 2540 | 2293 | 118 | 130 |
| 2d_4 | 4060 | 3699 | 166 | 196 |
| 2d_5 | 3680 | 3336 | 160 | 184 |
| 24h_1 | 5080 | 4639 | 205 | 236 |
| 24h_2 | 3590 | 3207 | 182 | 201 |
| 24h_3 | 3090 | 2827 | 138 | 125 |
| 24h_4 | 4680 | 4267 | 193 | 220 |
| 24h_5 | 5480 | 5004 | 221 | 256 |
| 7h_1 | 4910 | 4512 | 182 | 216 |
| 7h_2 | 2750 | 2492 | 125 | 133 |
| 7h_3 | 2540 | 2273 | 119 | 148 |
| 7h_4 | 3890 | 3575 | 138 | 177 |
| 7h_5 | 5470 | 4753 | 399 | 318 |
| 2h_1 | 2430 | 2268 | 84 | 79 |
| 2h_2 | 1200 | 1094 | 50 | 56 |
| 2h_3 | 766 | 696 | 33 | 37 |
| 2h_4 | 1530 | 1412 | 53 | 65 |
| 2h_5 | 2730 | 2551 | 92 | 87 |

**Table S3E:** Individual glycoform concentrations for the PK study after **intravenous injection** of **ST3 mAb1**Concentrations of individual glycoforms are calculated from the total antibody concentration determined by ELISA (total) and the glycosylation profiles (Table S10E). They are given in ng/mL separately for each time point and each animal (five biological replicates).

| Time point_animal | Total | G0F | G1F | G2FS1 | G2S2 | G2FS2 | H6N4F1S2 |
| --- | --- | --- | --- | --- | --- | --- | --- |
| 7d_1 | 871 | 19 | 6.3 | 39 | 45 | 756 | 5.1 |
| 7d_2 | 563 | 17 | 6.0 | 27 | 30 | 481 | 2.7 |
| 7d_3 | 530 | 11 | 5.4 | 30 | 26 | 455 | 3.0 |
| 7d_4 | 658 | 30 | 37 | 28 | 33 | 526 | 3.8 |
| 7d_5 | 729 | 17 | 6.3 | 26 | 38 | 637 | 4.1 |
| 4d_1 | 2370 | 116 | 45 | 142 | 124 | 1926 | 16.0 |
| 4d_2 | 1630 | 69 | 25 | 74 | 90 | 1361 | 10.1 |
| 4d_3 | 1450 | 74 | 29 | 89 | 78 | 1170 | 9.4 |
| 4d_4 | 2330 | 129 | 55 | 120 | 124 | 1887 | 15.6 |
| 4d_5 | 2320 | 111 | 41 | 106 | 120 | 1926 | 15.8 |
| 3d_1 | 3240 | 167 | 63 | 160 | 176 | 2655 | 19 |
| 3d_2 | 2520 | 110 | 45 | 111 | 143 | 2095 | 16 |
| 3d_3 | 3870 | 215 | 87 | 199 | 205 | 3138 | 27 |
| 3d_4 | 2780 | 120 | 47 | 111 | 155 | 2331 | 17 |
| 3d_5 | 3100 | 179 | 65 | 159 | 153 | 2524 | 19 |
| 2d_1 | 4560 | 371 | 143 | 279 | 241 | 3497 | 30 |
| 2d_2 | 3490 | 158 | 63 | 143 | 197 | 2901 | 28 |
| 2d_3 | 3140 | 239 | 105 | 224 | 161 | 2391 | 19 |
| 2d_4 | 3590 | 184 | 69 | 174 | 188 | 2948 | 28 |
| 2d_5 | 4220 | 239 | 92 | 179 | 234 | 3449 | 27 |
| 24h_1 | 6920 | 429 | 159 | 327 | 353 | 5607 | 44 |
| 24h_2 | 4990 | 248 | 98 | 227 | 276 | 4111 | 31 |
| 24h_3 | 5250 | 347 | 135 | 283 | 278 | 4173 | 34 |
| 24h_4 | 5150 | 253 | 98 | 183 | 280 | 4299 | 37 |
| 24h_5 | 6220 | 329 | 124 | 232 | 344 | 5145 | 45 |
| 7h_1 | 9280 | 580 | 226 | 591 | 479 | 7342 | 61 |
| 7h_2 | 8410 | 466 | 398 | 340 | 849 | 6306 | 50 |
| 7h_3 | 7860 | 482 | 179 | 419 | 394 | 6330 | 56 |
| 7h_4 | 8550 | 467 | 157 | 331 | 466 | 7072 | 58 |
| 7h_5 | 9190 | 521 | 195 | 408 | 472 | 7528 | 66 |
| 1h_1 | 12700 | 1029 | 364 | 735 | 603 | 9889 | 79 |
| 1h_2 | 10400 | 664 | 252 | 502 | 533 | 8373 | 76 |
| 1h_3 | 10300 | 649 | 247 | 526 | 565 | 8236 | 76 |
| 1h_4 | 10200 | 613 | 234 | 445 | 542 | 8290 | 76 |
| 1h_5 | 10200 | 690 | 256 | 530 | 553 | 8094 | 77 |
| 5min_1 | 12700 | 1194 | 447 | 1082 | 739 | 9165 | 73 |
| 5min_2 | 12100 | 809 | 318 | 601 | 638 | 9647 | 87 |
| 5min_3 | 11400 | 725 | 281 | 634 | 655 | 9029 | 76 |
| 5min_4 | 11100 | 637 | 251 | 448 | 602 | 9079 | 82 |
| 5min_5 | 12700 | 819 | 309 | 569 | 717 | 10189 | 97 |

**Table S3F:** Individual glycoform concentrations for the PK study after **subcutaneous injection** of **ST3 mAb1**Concentrations of individual glycoforms are calculated from the total antibody concentration determined by ELISA (total) and the glycosylation profiles (Table S10F). They are given in ng/mL separately for each time point and each animal (five biological replicates).

| Time point_animal | Total | G0F | G1F | G2FS1 | G2S2 | G2FS2 | H6N4F1S2 |
| --- | --- | --- | --- | --- | --- | --- | --- |
| 10d_1 | 188 | 4.5 | 0.3 | 12.8 | 8.0 | 160 | 2.0 |
| 10d_2 | 388 |  |  |  |  |  |  |
| 10d_3 | 518 | 12.1 | 8.2 | 30.4 | 22.7 | 440 | 4.3 |
| 10d_4 | 235 | 2.8 | 0.4 | 15.0 | 9.2 | 202 | 5.5 |
| 10d_5 | 441 | 8.5 | 3.6 | 21.3 | 21.6 | 382 | 4.3 |
| 7d_1 | 434 | 11.2 | 5.0 | 22.6 | 23.1 | 369 | 3.4 |
| 7d_2 | 754 | 19.1 | 9.4 | 41.5 | 41.9 | 640 | 2.4 |
| 7d_3 | 1200 | 28.0 | 20.4 | 46.5 | 60.0 | 1034 | 10.8 |
| 7d_4 | 713 | 22.0 | 12.6 | 38.6 | 35.2 | 600 | 4.3 |
| 7d_5 | 1000 | 36.7 | 20.2 | 61.2 | 49.5 | 825 | 7.2 |
| 4d_1 | 1310 | 51 | 27 | 76 | 63 | 1083 | 8.9 |
| 4d_2 | 1610 | 61 | 33 | 79 | 82 | 1343 | 11.6 |
| 4d_3 | 2400 | 98 | 50 | 115 | 123 | 1998 | 16.5 |
| 4d_4 | 1640 | 75 | 38 | 96 | 79 | 1340 | 11.6 |
| 4d_5 | 2260 | 71 | 19 | 151 | 100 | 1917 | 2.9 |
| 3d_1 | 1650 | 68 | 34 | 86 | 84 | 1366 | 12.3 |
| 3d_2 | 3010 | 108 | 60 | 85 | 154 | 2580 | 22.1 |
| 3d_3 | 2860 | 125 | 69 | 151 | 139 | 2356 | 20.7 |
| 3d_4 | 2030 | 91 | 49 | 117 | 97 | 1661 | 14.9 |
| 3d_5 | 2870 | 91 | 48 | 94 | 155 | 2461 | 20.7 |
| 2d_1 | 3210 | 138 | 73 | 134 | 166 | 2675 | 24.5 |
| 2d_2 | 2880 | 124 | 63 | 135 | 152 | 2384 | 21.4 |
| 2d_3 | 4170 | 204 | 109 | 216 | 213 | 3397 | 30.7 |
| 2d_4 | 2670 | 124 | 64 | 140 | 130 | 2192 | 20.4 |
| 2d_5 | 3440 | 162 | 83 | 194 | 189 | 2801 | 11.8 |
| 24h_1 | 4490 | 203 | 110 | 203 | 250 | 3688 | 35.9 |
| 24h_2 | 4160 | 164 | 87 | 154 | 221 | 3502 | 30.9 |
| 24h_3 | 6080 | 258 | 142 | 197 | 345 | 5091 | 46.5 |
| 24h_4 | 2800 | 115 | 64 | 98 | 150 | 2351 | 21.2 |
| 24h_5 | 4350 | 259 | 138 | 227 | 203 | 3491 | 32.3 |
| 7h_1 | 2650 | 174 | 90 | 174 | 117 | 2077 | 17.6 |
| 7h_2 | 3300 | 212 | 109 | 240 | 159 | 2560 | 21.7 |
| 7h_3 | 4940 | 282 | 151 | 275 | 235 | 3960 | 37.2 |
| 7h_4 | 1970 | 85 | 49 | 88 | 106 | 1631 | 11.3 |
| 7h_5 | 4010 | 195 | 106 | 160 | 209 | 3309 | 30.8 |
| 2h_1 | 1250 | 69 | 36 | 72 | 58 | 1006 | 8.3 |
| 2h_2 | 2330 | 107 | 49 | 130 | 115 | 1913 | 15.9 |
| 2h_3 | 1710 | 99 | 48 | 96 | 83 | 1373 | 11.5 |
| 2h_4 | 561 | 32 | 16 | 31 | 27 | 451 | 4.3 |
| 2h_5 | 1140 | 53 | 21 | 63 | 54 | 943 | 7.1 |

**Table S3G:** Individual glycoform concentrations for the PK study after **subcutaneous injection** of **CHO mAb2**Concentrations of individual glycoforms are calculated from the total antibody concentration determined by ELISA (total) and the glycosylation profiles (Table S10G). They are given in ng/mL separately for each time point and each animal (five biological replicates). Actual sampling times are gives as well.

| Animal_ time point | Sampling time [min] | Total | Man5 | G0F-N | G0 | G0F | G1 | G1F | G2F |
| --- | --- | --- | --- | --- | --- | --- | --- | --- | --- |
| 101F1_2h | 120 | 4044 | 39 | 77 | 53 | 1720 | 42 | 1780 | 334 |
| 102F1_2h | 120 | 5104 | 49 | 96 | 65 | 2170 | 49 | 2253 | 422 |
| 103F1_2h | 120 | 6505 | 62 | 124 | 86 | 2777 | 58 | 2866 | 532 |
| 104F1_2h | 120 | 7227 | 69 | 137 | 91 | 3084 | 67 | 3189 | 589 |
| 105F1_2h | 120 | 7416 | 70 | 148 | 93 | 3156 | 63 | 3282 | 605 |
| 101F1_7h | 421 | 10890 | 111 | 219 | 144 | 4622 | 122 | 4760 | 912 |
| 102F1_7h | 420 | 10220 | 104 | 215 | 137 | 4335 | 88 | 4485 | 857 |
| 103F1_7h | 419 | 12450 | 128 | 241 | 161 | 5295 | 100 | 5481 | 1043 |
| 104F1_7h | 419 | 12440 | 128 | 253 | 169 | 5287 | 100 | 5459 | 1044 |
| 105F1_7h | 420 | 12420 | 126 | 248 | 175 | 5272 | 101 | 5445 | 1053 |
| 101F1_24h | 1430 | 16670 | 164 | 333 | 226 | 7125 | 178 | 7255 | 1389 |
| 102F1_24h | 1428 | 11300 | 112 | 229 | 146 | 4810 | 90 | 4962 | 951 |
| 103F1_24h | 1424 | 14990 | 153 | 299 | 210 | 6382 | 122 | 6559 | 1266 |
| 104F1_24h | 1424 | 15450 | 135 | 310 | 205 | 6591 | 159 | 6756 | 1295 |
| 105F1_24h | 1422 | 15640 | 151 | 311 | 211 | 6633 | 151 | 6862 | 1321 |
| 101F1_2d | 2892 | 20450 | 198 | 405 | 275 | 8686 | 186 | 8967 | 1733 |
| 102F1_2d | 2890 | 15180 | 147 | 299 | 198 | 6475 | 139 | 6661 | 1260 |
| 103F1_2d | 2888 | 18190 | 185 | 362 | 253 | 7733 | 197 | 7922 | 1537 |
| 104F1_2d | 2897 | 17840 | 142 | 371 | 249 | 7607 | 160 | 7799 | 1513 |
| 105F1_2d | 2895 | 17320 | 170 | 351 | 238 | 7338 | 184 | 7561 | 1479 |
| 101F1_3d | 4341 | 19840 | 187 | 401 | 277 | 8425 | 209 | 8630 | 1712 |
| 102F1_3d | 4339 | 14880 | 149 | 303 | 207 | 6325 | 119 | 6511 | 1267 |
| 103F1_3d | 4335 | 20590 | 203 | 419 | 297 | 8755 | 216 | 8936 | 1764 |
| 104F1_3d | 4334 | 19390 | 146 | 389 | 272 | 8216 | 183 | 8514 | 1671 |
| 105F1_3d | 4333 | 16510 | 154 | 338 | 232 | 7024 | 193 | 7171 | 1398 |
| 101F1_4d | 5777 | 19790 | 182 | 403 | 276 | 8412 | 213 | 8628 | 1676 |
| 102F1_4d | 5774 | 16500 | 159 | 336 | 224 | 7016 | 182 | 7180 | 1404 |
| 103F1_4d | 5771 | 18970 | 182 | 390 | 261 | 8040 | 195 | 8297 | 1605 |
| 104F1_4d | 5771 | 18590 | 134 | 383 | 249 | 7928 | 209 | 8132 | 1556 |
| 105F1_4d | 5768 | 16730 | 150 | 338 | 226 | 7137 | 189 | 7294 | 1396 |
| 101F1_7d | 10092 | 17320 | 146 | 346 | 232 | 7348 | 155 | 7642 | 1451 |
| 102F1_7d | 10090 | 18090 | 163 | 356 | 241 | 7696 | 205 | 7897 | 1532 |
| 103F1_7d | 10087 | 19420 | 175 | 393 | 266 | 8248 | 188 | 8492 | 1658 |
| 104F1_7d | 10087 | 18430 | 122 | 364 | 248 | 7846 | 163 | 8120 | 1566 |
| 105F1_7d | 10084 | 15490 | 129 | 306 | 215 | 6601 | 142 | 6794 | 1302 |
| 101F1_10d | 14412 | 16720 | 134 | 329 | 220 | 7108 | 178 | 7332 | 1418 |
| 102F1_10d | 14411 | 16650 | 136 | 329 | 223 | 7085 | 187 | 7296 | 1394 |
| 103F1_10d | 14409 | 18330 | 142 | 355 | 238 | 7805 | 180 | 8069 | 1541 |
| 104F1_10d | 14410 | 15740 | 91 | 305 | 206 | 6695 | 158 | 6959 | 1326 |
| 105F1_10d | 14408 | 14830 | 112 | 300 | 201 | 6319 | 154 | 6491 | 1252 |
| 101F1_14d | 20188 | 11130 | 60 | 211 | 142 | 4773 | 91 | 4930 | 923 |
| 102F1_14d | 20186 | 12560 | 83 | 243 | 166 | 5362 | 104 | 5555 | 1048 |
| 103F1_14d | 20184 | 16900 | 120 | 323 | 225 | 7191 | 153 | 7479 | 1409 |
| 104F1_14d | 20183 | 14390 | 76 | 282 | 182 | 6129 | 123 | 6365 | 1233 |
| 105F1_14d | 20180 | 13040 | 86 | 254 | 176 | 5551 | 112 | 5768 | 1092 |
| 101F1_21d | 30261 | 3586 | 17 | 66 | 45 | 1531 | 33 | 1590 | 303 |
| 102F1_21d | 30260 | 3429 | 17 | 64 | 39 | 1459 | 29 | 1528 | 294 |
| 103F1_21d | 30258 | 12560 | 67 | 238 | 159 | 5386 | 109 | 5553 | 1048 |
| 104F1_21d | 30259 | 11520 | 66 | 221 | 164 | 4887 | 113 | 5094 | 976 |
| 105F1_21d | 30257 | 7319 | 34 | 131 | 91 | 3170 | 66 | 3226 | 600 |
| 101F1_28d | 40348 | 2197 | 7.2 | 46 | 27 | 942 | 20 | 966 | 187 |
| 102F1_28d | 40347 | 477 |  |  |  |  |  |  |  |
| 103F1_28d | 40346 | 8857 | 38 | 164 | 108 | 3801 | 83 | 3945 | 717 |
| 104F1_28d | 40347 | 9810 | 32 | 178 | 122 | 4211 | 95 | 4378 | 794 |
| 105F1_28d | 40345 | 5122 | 19 | 95 | 68 | 2229 | 48 | 2247 | 417 |
| 101F1_35d | 50418 | 1782 | 7.1 | 35 | 22 | 759 | 18 | 789 | 152 |
| 102F1_35d | 50417 | 307 |  |  |  |  |  |  |  |
| 103F1_35d | 50414 | 5617 | 21 | 101 | 71 | 2387 | 50 | 2508 | 479 |
| 104F1_35d | 50414 | 6090 | 22 | 109 | 78 | 2607 | 51 | 2712 | 512 |
| 105F1_35d | 50412 | 3543 | 16 | 67 | 49 | 1551 | 30 | 1540 | 290 |
| 101F1_42d | 60507 | 1351 | 4.0 | 26 | 17 | 572 | 18 | 599 | 115 |
| 102F1_42d | 60505 | 255 |  |  |  |  |  |  |  |
| 103F1_42d | 60501 | 3621 | 12 | 66 | 43 | 1530 | 31 | 1630 | 309 |
| 104F1_42d | 60502 | 6035 | 19 | 113 | 82 | 2564 | 54 | 2693 | 509 |
| 105F1_42d | 60499 | 2286 | 8.0 | 45 | 30 | 1015 | 18 | 985 | 185 |

**Table S3H:** Individual glycoform concentrations for the PK study after **subcutaneous injection** of **ST3 mAb2**

Concentrations of individual glycoforms are calculated from the total antibody concentration determined by ELISA (total) and the glycosylation profiles (Table S10H). They are given in ng/mL separately for each time point and each animal (five biological replicates). Actual sampling times are gives as well.

| Animal_time point | Sampling time [min] | Total | Man5 | G1S-N | G1FS-N | G2F | G2S1 | G2FS1 | G2S2 | G2FS2 |
| --- | --- | --- | --- | --- | --- | --- | --- | --- | --- | --- |
| 201F1_2h | 120 | 5100 | 33 | 14 | 95 | 133 | 31 | 1388 | 66 | 3339 |
| 202F1_2h | 120 | 5060 | 32 | 13 | 98 | 144 | 31 | 1433 | 65 | 3245 |
| 203F1_2h | 120 | 7910 | 52 | 18 | 155 | 179 | 46 | 2102 | 105 | 5253 |
| 204F1_2h | 120 | 7060 | 50 | 20 | 141 | 169 | 40 | 1891 | 94 | 4655 |
| 205F1_2h | 120 | 5450 | 35 | 16 | 101 | 132 | 31 | 1456 | 73 | 3606 |
| 201F1_7h | 418 | 11000 | 81 | 26 | 222 | 308 | 67 | 3088 | 155 | 7053 |
| 202F1_7h | 417 | 11950 | 87 | 33 | 259 | 378 | 76 | 3509 | 172 | 7435 |
| 203F1_7h | 418 | 14210 | 99 | 36 | 280 | 391 | 84 | 4029 | 192 | 9099 |
| 204F1_7h | 417 | 17840 | 132 | 47 | 355 | 511 | 110 | 5122 | 247 | 11316 |
| 205F1_7h | 417 | 14290 | 105 | 40 | 290 | 384 | 90 | 4007 | 191 | 9184 |
| 201F1_24h | 1420 | 17190 | 116 | 41 | 345 | 542 | 107 | 5078 | 231 | 10730 |
| 202F1_24h | 1418 | 17700 | 106 | 45 | 351 | 508 | 103 | 5025 | 235 | 11327 |
| 203F1_24h | 1418 | 17060 | 123 | 46 | 348 | 492 | 102 | 4860 | 237 | 10852 |
| 204F1_24h | 1417 | 21550 | 163 | 55 | 434 | 655 | 133 | 6285 | 289 | 13536 |
| 205F1_24h | 1416 | 17070 | 117 | 43 | 332 | 500 | 104 | 4895 | 231 | 10849 |
| 201F1_2d | 2892 | 18860 | 128 | 51 | 388 | 535 | 116 | 5409 | 255 | 11979 |
| 202F1_2d | 2891 | 19410 | 115 | 49 | 380 | 598 | 119 | 5700 | 262 | 12186 |
| 203F1_2d | 2891 | 18610 | 128 | 49 | 376 | 518 | 113 | 5320 | 260 | 11844 |
| 204F1_2d | 2890 | 21650 | 168 | 60 | 434 | 630 | 127 | 6206 | 305 | 13719 |
| 205F1_2d | 2888 | 18780 | 124 | 47 | 364 | 555 | 122 | 5484 | 261 | 11823 |
| 201F1_3d | 4331 | 18340 | 104 | 44 | 376 | 516 | 112 | 5252 | 253 | 11682 |
| 202F1_3d | 4330 | 19260 | 103 | 46 | 382 | 537 | 122 | 5438 | 263 | 12370 |
| 203F1_3d | 4329 | 19230 | 125 | 49 | 372 | 577 | 126 | 5582 | 261 | 12138 |
| 204F1_3d | 4327 | 22120 | 152 | 56 | 443 | 645 | 138 | 6312 | 315 | 14059 |
| 205F1_3d | 4326 | 18670 | 123 | 48 | 381 | 554 | 120 | 5428 | 264 | 11753 |
| 201F1_4d | 5767 | 19350 | 99 | 43 | 386 | 587 | 123 | 5664 | 273 | 12174 |
| 202F1_4d | 5766 | 18090 | 87 | 44 | 366 | 537 | 116 | 5263 | 251 | 11427 |
| 203F1_4d | 5766 | 18870 | 129 | 47 | 375 | 526 | 122 | 5339 | 265 | 12067 |
| 204F1_4d | 5765 | 20440 | 144 | 61 | 411 | 626 | 133 | 5891 | 325 | 12848 |
| 205F1_4d | 5763 | 19490 | 128 | 49 | 377 | 600 | 129 | 5716 | 269 | 12222 |
| 201F1_7d | 10081 | 17000 | 85 | 46 | 359 | 510 | 106 | 4939 | 261 | 10694 |
| 202F1_7d | 10079 | 15920 | 81 | 38 | 329 | 511 | 102 | 4775 | 213 | 9871 |
| 203F1_7d | 10078 | 17550 | 105 | 44 | 342 | 546 | 115 | 5173 | 243 | 10981 |
| 204F1_7d | 10077 | 19010 | 126 | 47 | 384 | 607 | 129 | 5612 | 260 | 11846 |
| 205F1_7d | 10076 | 17880 | 98 | 48 | 350 | 499 | 110 | 5051 | 245 | 11479 |
| 201F1_20d | 14405 | 15960 | 70 | 38 | 322 | 454 | 99 | 4589 | 221 | 10167 |
| 202F1_20d | 14404 | 15340 | 66 | 37 | 308 | 449 | 101 | 4443 | 212 | 9722 |
| 203F1_20d | 14404 | 16770 | 91 | 41 | 342 | 502 | 103 | 4894 | 227 | 10569 |
| 204F1_20d | 14403 | 17310 | 102 | 45 | 351 | 537 | 115 | 5043 | 242 | 10875 |
| 205F1_20d | 14402 | 16500 | 83 | 39 | 328 | 460 | 99 | 4710 | 221 | 10560 |
| 201F1_14d | 20177 | 14110 | 50 | 32 | 269 | 375 | 90 | 3975 | 192 | 9127 |
| 202F1_14d | 20175 | 12980 | 51 | 30 | 252 | 390 | 76 | 3783 | 179 | 8219 |
| 203F1_14d | 20174 | 15920 | 81 | 38 | 321 | 428 | 98 | 4453 | 219 | 10282 |
| 204F1_14d | 20172 | 17990 | 96 | 42 | 352 | 559 | 121 | 5277 | 254 | 11289 |
| 205F1_14d | 20170 | 14600 | 65 | 33 | 285 | 419 | 94 | 4200 | 201 | 9303 |
| 201F1_21d | 30256 | 10670 | 40 | 23 | 205 | 292 | 76 | 2954 | 152 | 6929 |
| 202F1_21d | 30256 | 10500 | 39 | 20 | 204 | 278 | 70 | 2879 | 145 | 6866 |
| 203F1_21d | 30257 | 12590 | 51 | 28 | 237 | 339 | 73 | 3545 | 170 | 8146 |
| 204F1_21d | 30256 | 13230 | 62 | 31 | 252 | 379 | 94 | 3774 | 188 | 8449 |
| 205F1_21d | 30258 | 5720 | 23 | 14 | 106 | 147 | 47 | 1523 | 81 | 3781 |
| 201F1_28d | 40343 | 10660 | 44 | 29 | 257 | 372 | 88 | 3082 | 162 | 6627 |
| 202F1_28d | 40343 | 10100 | 37 | 21 | 197 | 269 | 71 | 2785 | 138 | 6583 |
| 203F1_28d | 40343 | 11640 | 39 | 26 | 215 | 302 | 65 | 3149 | 154 | 7690 |
| 204F1_28d | 40342 | 12550 | 49 | 32 | 238 | 342 | 73 | 3471 | 169 | 8176 |
| 205F1_28d | 40341 | 90 |  |  |  |  |  |  |  |  |
| 201F1_35d | 50410 | 8780 | 28 | 18 | 171 | 261 | 67 | 2546 | 128 | 5560 |
| 202F1_35d | 50409 | 8560 | 26 | 20 | 162 | 226 | 60 | 2383 | 125 | 5558 |
| 203F1_35d | 50408 | 9560 | 33 | 20 | 182 | 256 | 68 | 2660 | 134 | 6207 |
| 204F1_35d | 50407 | 10690 | 45 | 27 | 208 | 292 | 73 | 2958 | 149 | 6938 |
| 205F1_35d | 50406 | BLQ |  |  |  |  |  |  |  |  |
| 201F1_42d | 60497 | 7070 | 24 | 13 | 131 | 191 | 50 | 1927 | 104 | 4631 |
| 202F1_42d | 60495 | 6650 | 26 | 14 | 127 | 175 | 52 | 1811 | 94 | 4351 |
| 203F1_42d | 60495 | 8520 | 34 | 20 | 166 | 225 | 62 | 2336 | 123 | 5554 |
| 204F1_42d | 60493 | 8910 | 36 | 21 | 171 | 225 | 69 | 2396 | 126 | 5865 |
| 205F1_42d | 60492 | BLQ |  |  |  |  |  |  |  |  |

**Table S12: Glycoform profiles for the mAbs prior to injection**

mAb profiles were measured in relevant matrix by spiking into blank serum at 10 mg/L (three full technical replicates). These reference profiles were measured at the same time as the PK samples to avoid batch effects, hence the distinction between IV (intravenous) and SC (subcutaneous) experiments for mAb1.

**A: CHO mAb1 IV**

| Man5 | G0F-N | G0 | G0F | G1 | G1F | G2F |
| --- | --- | --- | --- | --- | --- | --- |
| 2.26% | 5.08% | 3.86% | 61.42% | 1.35% | 23.82% | 2.21% |
| 2.38% | 5.06% | 3.78% | 61.31% | 1.37% | 23.86% | 2.24% |
| 2.39% | 6.30% | 3.96% | 60.06% | 1.27% | 23.79% | 2.23% |

**B: CHO mAb1 SC**

| Man5 | G0F-N | G0 | G0F | G1 | G1F | G2F |
| --- | --- | --- | --- | --- | --- | --- |
| 0.79% | 4.32% | 3.99% | 63.10% | 1.24% | 24.49% | 2.08% |
| 1.06% | 6.55% | 4.38% | 61.68% | 1.17% | 23.20% | 1.96% |
| 0.81% | 5.03% | 3.98% | 62.91% | 1.25% | 24.02% | 1.99% |

**C: M5 mAb1 IV**

| Man5 | G0F | G1F |
| --- | --- | --- |
| 90.98% | 4.32% | 4.70% |
| 90.72% | 4.32% | 4.96% |
| 91.60% | 4.24% | 4.16% |

**D: M5 mAb1 SC**

| Man5 | G0F | G1F |
| --- | --- | --- |
| 91.35% | 3.92% | 4.72% |
| 92.76% | 3.71% | 3.53% |
| 87.36% | 4.95% | 7.69% |

**E: ST3 mAb1 IV**

| G0F | G1F | G2FS1 | G2S2 | G2FS2 | H6N4F1S2 |
| --- | --- | --- | --- | --- | --- |
| 6.41% | 2.45% | 4.60% | 5.22% | 80.60% | 0.72% |
| 6.58% | 2.85% | 4.82% | 4.91% | 80.12% | 0.72% |
| 6.28% | 2.37% | 4.55% | 5.02% | 81.11% | 0.67% |

**F: ST3 mAb1 SC**

| G0F | G1F | G2FS1 | G2S2 | G2FS2 | H6N4F1S2 |
| --- | --- | --- | --- | --- | --- |
| 6.54% | 2.49% | 5.64% | 4.73% | 79.89% | 0.71% |
| 6.91% | 2.68% | 6.46% | 4.79% | 78.46% | 0.69% |
| 7.37% | 2.86% | 6.30% | 4.52% | 78.27% | 0.68% |

**G: CHO_mAb2**

| Man5 | G0F-N | G0 | G0F | G1 | G1F | G2F |
| --- | --- | --- | --- | --- | --- | --- |
| 1.08% | 1.98% | 1.29% | 42.20% | 0.84% | 44.32% | 8.29% |
| 1.04% | 1.98% | 1.29% | 42.64% | 0.87% | 43.87% | 8.31% |
| 1.07% | 2.01% | 1.40% | 42.59% | 1.01% | 43.63% | 8.29% |

**H: ST3_mAb2**

| Man5 | G2F | G1S-N | G1FS-N | G2S1 | G2FS1 | G2S2 | G2FS2 |
| --- | --- | --- | --- | --- | --- | --- | --- |
| 1.64% | 3.08% | 0.42% | 3.32% | 0.65% | 28.13% | 2.15% | 60.61% |
| 1.99% | 2.82% | 0.44% | 3.47% | 0.76% | 27.65% | 2.40% | 60.46% |
| 1.78% | 3.33% | 0.44% | 3.45% | 0.74% | 29.33% | 2.24% | 58.70% |
